# Amyloid-β-Induced Dendritic Spine Elimination Requires Ca^2+^-Permeable AMPA Receptors, AKAP-Calcineurin-NFAT Signaling, and the NFAT Target Gene Mdm2

**DOI:** 10.1523/ENEURO.0175-23.2024

**Published:** 2024-03-06

**Authors:** Tyler P. Martinez, Matthew E. Larsen, Emily Sullivan, Kevin M. Woolfrey, Mark L. Dell’Acqua

**Affiliations:** ^1^Pharmacology PhD Program, University of Colorado Anschutz Medical Campus, Aurora, Colorado 80045; ^2^Department of Pharmacology, University of Colorado Anschutz Medical Campus, Aurora, Colorado 80045; ^3^Linda Crnic Institute for Down Syndrome, University of Colorado Anschutz Medical Campus, Aurora, Colorado 80045; ^4^Neuroscience PhD Program, University of Colorado Anschutz Medical Campus, Aurora, Colorado 80045; ^5^Neurotechnology Center, University of Colorado Anschutz Medical Campus, Aurora, Colorado 80045; ^6^Alzheimer’s and Cognition Center, University of Colorado Anschutz Medical Campus, Aurora, Colorado 80045

**Keywords:** AKAP, amyloid-β, Ca^2+^-permeable AMPA receptor, calcineurin, dendritic spine, NFAT

## Abstract

Alzheimer's disease (AD) is associated with brain accumulation of synaptotoxic amyloid-β (Aβ) peptides produced by the proteolytic processing of amyloid precursor protein (APP). Cognitive impairments associated with AD correlate with dendritic spine and excitatory synapse loss, particularly within the hippocampus. In rodents, soluble Aβ oligomers (Aβo) impair hippocampus-dependent learning and memory, promote dendritic spine loss, inhibit NMDA-type glutamate receptor (NMDAR)-dependent long-term potentiation (LTP), and promote synaptic depression (LTD), at least in part through activation of the Ca^2+^-CaM-dependent phosphatase calcineurin (CaN). Yet, questions remain regarding Aβ-dependent postsynaptic CaN signaling specifically at the synapse to mediate its synaptotoxicity. Here, we use pharmacologic and genetic approaches to demonstrate a role for postsynaptic signaling via A kinase-anchoring protein 150 (AKAP150)-scaffolded CaN in mediating Aβ-induced dendritic spine loss in hippocampal neurons from rats and mice of both sexes. In particular, we found that Ca^2+^-permeable AMPA-type glutamate receptors (CP-AMPARs), which were previously shown to signal through AKAP-anchored CaN to promote both LTD and Aβ-dependent inhibition of LTP, are also required upstream of AKAP-CaN signaling to mediate spine loss via overexpression of APP containing multiple mutations linked to familial early-onset AD (FAD) and increased Aβ production. In addition, we found that the CaN-dependent nuclear factor of activated T-cells (NFAT) transcription factors is required downstream to promote Aβ-mediated dendritic spine loss. Finally, we identified the E3-ubiquitin ligase Mdm2, which was previously linked to LTD and developmental synapse elimination, as a downstream NFAT target gene upregulated by Aβ whose enzymatic activity is required for Aβ-mediated spine loss.

## Graphical abstract

**Figure:**
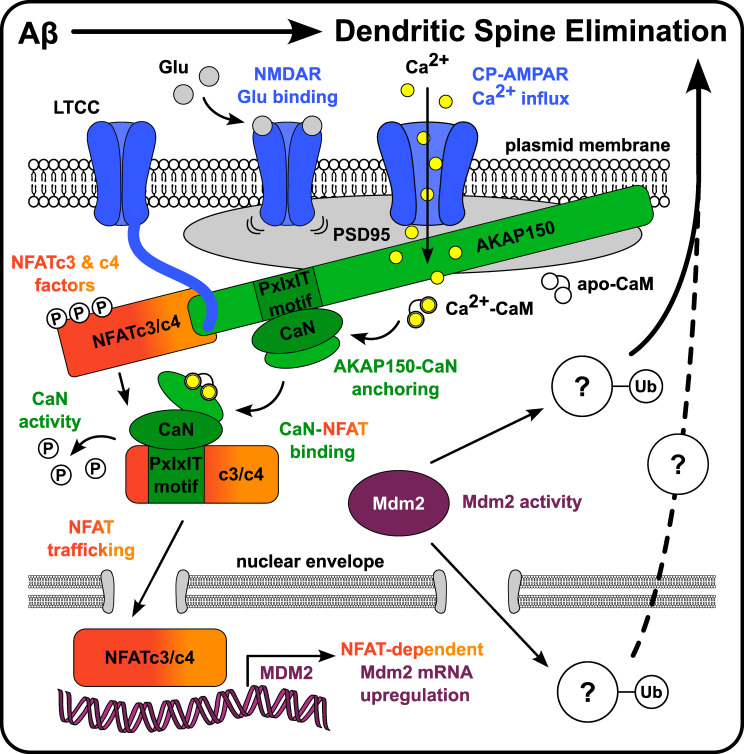
Visual Overview

## Significance Statement

Impaired hippocampal function and synapse loss are hallmarks of AD linked to Aβ oligomers. Aβ exposure acutely blocks hippocampal LTP and enhances LTD and chronically leads to dendritic spine synapse loss. In particular, Aβ hijacks normal plasticity mechanisms, biasing them toward synapse weakening/elimination, with previous studies broadly linking CaN phosphatase signaling to this synaptic dysfunction. However, we do not understand how Aβ engages signaling specifically at synapses. Here we elucidate a synapse-to-nucleus signaling pathway coordinated by the postsynaptic scaffold protein AKAP150 that is activated by Ca^2+^ influx through CP-AMPARs and transduced to nucleus by CaN-NFAT signaling to transcriptionally upregulate the E3-ubiquitin ligase Mdm2 that is required for Aβ-mediated spine loss. These findings identify Mdm2 as potential therapeutic target for AD.

## Introduction

AD is the most prevalent neurodegenerative dementia and is characterized by impairment of learning, memory, and other cognitive functions that principally originates from progressive synapse loss across the cortex and hippocampus (Selkoe and Hardy, 2016). Although most cases of AD are sporadic, every mutation known to segregate with familial early-onset AD (FAD) confers increased production of neurotoxic amyloid-β (Aβ) peptides by altering proteolytic processing of APP (O'Brien and Wong, 2011). Thirty years of evidence have established the accumulation of Aβ oligomers (Aβo) in AD pathogenesis, and 42-amino acid oligomers (Aβ_42_) appear especially synaptotoxic (Li and Selkoe, 2020). However, given that monoclonal antibody-based therapeutics directly targeting Aβ have shown only limited promise to date (Cummings et al., 2022), investigation of signaling downstream of Aβ is undoubtedly also crucial for the development of novel disease-modifying AD treatments.

Prior studies in rodents demonstrated that soluble Aβo (1) impair hippocampus-dependent learning and memory (Shankar et al., 2008), (2) drive hippocampal excitatory synapse elimination and dendritic spine loss (Hsieh et al., 2006; Shankar et al., 2007, 2008; Wei et al., 2010), and (3) impair long-term potentiation (LTP) and promote LTD (Chen et al., 2002; Hsieh et al., 2006; Shankar et al., 2007, 2008). Notably, pharmacologic approaches have implicated NMDARs (Shankar et al., 2007; Wei et al., 2010; Kessels et al., 2013) and the phosphatase calcineurin (CaN) (Chen et al., 2002; Hsieh et al., 2006; Shankar et al., 2007; Reese et al., 2008; Wu et al., 2010, 2012; Zhao et al., 2010) in Aβ-induced hippocampal synaptic dysfunction and synapse loss in manners that are also consistent with their established roles in normal synaptic plasticity and synapse elimination. Although oversimplified, NMDARs are required for multiple forms of LTP and LTD (Luscher and Malenka, 2012), and CaN constrains NMDAR-dependent LTP and promotes LTD (Mulkey et al., 1994; Wang and Kelly, 1996; Zeng et al., 2001; Sanderson et al., 2012). More specifically, CaN is required for removal of AMPA-type glutamate receptors (AMPARs) from synapses during functional NMDAR-LTD (Beattie et al., 2000; Lee et al., 2000; Man et al., 2007; Sanderson et al., 2012), and for actin depolymerization via slingshot–cofilin signaling during structural LTD, which can ultimately promote spine collapse and synapse loss (Zhou et al., 2004; Wang et al., 2005). Nevertheless, our understanding of NMDAR- and CaN-dependent signaling in Aβ-induced synapse elimination remains incomplete.

Although NMDAR Ca^2+^ influx historically plays a critical role in LTP and LTD (Luscher and Malenka, 2012) and Aβ potently impairs NMDAR Ca^2+^ influx (Sinnen et al., 2016), recent studies indicate that nonionotropic conformational NMDAR signaling may be sufficient for functional and structural LTD (Nabavi et al., 2013; Stein et al., 2015) and for Aβ-induced dendritic spine loss (Kessels et al., 2013; Birnbaum et al., 2015). However, Ca^2+^-permeable AMPARs (CP-AMPARs) lacking GluA2 subunits have also been shown to play prominent roles in NMDAR-dependent LTP and LTD (Purkey and Dell'Acqua, 2020), and recently also in Aβ-mediated inhibition of LTP (Sanderson et al., 2021), yet their role in Aβ-induced spine loss is unexplored. Moreover, the removal of both Ca^2+^-impermeable (CI)-AMPAR and CP-AMPAR from synapses during LTD and Aβ-mediated inhibition of LTP is tightly coordinated by CaN anchoring to the postsynaptic scaffold A kinase-anchoring protein 150 (AKAP150) (Bhattacharyya et al., 2009; Jurado et al., 2010; Sanderson et al., 2012, 2016, 2021); however, local postsynaptic CaN signaling has not been explored in Aβ-induced spine loss. Lastly, while some prior studies of Aβ-induced spine loss implicated CaN-dependent activation of the nuclear factor of activated T-cells (NFAT) family of transcription factors (Wu et al., 2010; Hudry et al., 2012), a process that is also coordinated by AKAP150 (Oliveria et al., 2007; Zhang and Shapiro, 2012; Murphy et al., 2014), the manipulations employed to inhibit CaN-NFAT interactions in these prior studies also disrupt interactions with many other CaN interacting proteins (Li et al., 2011; Goldman et al., 2014). Furthermore, the NFAT transcriptional targets required for Aβ-induced spine loss have not been investigated.

Here, using a range of pharmacologic and genetic manipulations we interrogate the postsynaptic signaling mechanisms required for Aβ-mediated spine loss in cultured hippocampal neurons. We examine both ionotropic and conformational NMDAR signaling; we test the involvement of CP-AMPARs, CaN phosphatase activity, AKAP150-anchoring of CaN, and NFAT transcription factors; and importantly we implicate the NFAT transcriptional target and E3-ubiquitin ligase, murine double-minute 2 (Mdm2) as being necessary for Aβ-induced dendritic spine loss.

## Materials and Methods

A detailed description of key reagents, plasmids, experimental animal strains, and software used in this study can be found in Table 1.

**Table 1.** Key reagents and resources

Reagent or resource	Source	Identifier
**Small molecules, toxins, and peptides**		
D,L-AP5	Tocris Bioscience	catalog #0105
(+)-MK-801	Tocris Bioscience	catalog #0924
Nimodipine (nim)	Millipore	catalog #N149
NASPM trihydrochloride	Tocris Bioscience	catalog #2766
Cyclosporin A (CsA)	Tocris Bioscience	catalog #1101
Tacrolimus (FK506)	Tocris Bioscience	catalog #3631
Nutlin-3, racemic	Millipore	catalog #444143
Tetrodotoxin (TTX)	Tocris Bioscience	catalog #1078
Amyloid-beta (1–42) peptide	AnaSpec	catalog #20276
**Antibodies**		
Monoclonal mouse anti-MDM2	Santa Cruz Biotechnologies	catalog #sc-965 [SMP14]
Monoclonal mouse anti-actin	Millipore	catalog #MAB1501 [C4]
Monoclonal mouse anti-GAPDH	GeneTex	catalog #GTX627408 [GT239]
Monoclonal mouse anti-PSD95	BioLegend	catalog #810401 [K28/43]
Monoclonal mouse anti-ubiquitin	Santa Cruz Biotechnologies	catalog #sc-8017 [P4D1]
**Recombinant plasmid DNA**		
pCAG-[ctMCS] (custom “pCAGGS” backbone)	gift from Dr. Matthew Kennedy	
pCAG-hAPP695(Swe-Ind)	Addgene	catalog #30145
pCAG-hAPP695(M596V)	This publication	
pCMV-SGFP2-hNFATc3	Wild et al., 2019	
pCMV-SGFP2-mNFATc3	This publication	
pCMV-SGFP2-mNFATc3(PSAQAT)	This publication	
pCMV-SGFP2-mNFATc4	This publication	
pmU6-[shRNA]/“pSilencer 1.0-U6”	Ambion	catalog #AM7207
pmU6-NFATc3shRNA	This publication	
pmU6-NFATc4shRNA	This publication	
pmU6-[shRNA]-hSyn-mCh (“pFSW” lenti shuttle)	gift from Dr. Jason Aoto	
pmU6-NFATc3shRNA-hSyn-mCh	This publication	
pmU6-NFATc4shRNA-hSyn-mCh	This publication	
**Experimental models and strains**		
Sprague Dawley rats	Charles River Laboratories	catalog #Crl:CD(SD)
AKAP150ΔPIX mice (allele: AKAP5 tm1Mdaq)	Sanderson et al., 2012	RRID MGI_5448682
C57BL/6J mice	Jackson Laboratories	RRID IMSR_JAX:000664
**Software**		
SlideBook 5.5–6.0	3i-Intelligent Imaging Innovations	https://www.intelligent-imaging.com/slidebook
Fiji/ImageJ2	LOCI, University of Wisconsin-Madison	https://imagej.net/software/fiji/
Prism 6–8	GraphPad	https://www.graphpad.com/
blindanalysis v1.0/blindrename.pl	Jim Salter	https://github.com/jimsalterjrs/blindanalysis

### Dissociated primary hippocampal culture

All animal procedures were performed in accordance with the National Institutes of Health (NIH)–United States Public Health Service guidelines and with the approval of the University of Colorado Anschutz Institutional Animal Care and Use Committee. Rat hippocampi were resected from male and female P0–P1 Sprague Dawley neonates, dissociated with papain, and plated onto poly-D-lysine (PDL)-coated 18 mm no. 1.0 coverglass for imaging (1.8 × 10^6^ cells/12-well) or directly onto PDL-coated cell culture plates for qRT-PCR (2.2 × 10^6^ cells/6-well) and immunoblotting (1.1 × 10^6^ cells/6 cm dish). Cells were plated in MEM (Gibco) supplemented with L-glutamine, FBS, and Pen-Strep; maintained at 37°C and 5% CO_2_; and media was fully exchanged for Neurobasal-A (Gibco) supplemented with GlutaMAX (Gibco), B-27 (Gibco), and Pen-Strep at 1 DIV. Rat cells were fed every 5–6 d thereafter by exchanging one-half of the conditioned media for fresh supplemented media, and mitotic inhibitors (uridine + fluoro-deoxyuridine; Sigma) were added at 5–6 DIV. Similarly, mouse hippocampi were resected from male and female P0–P2 WT and AKAP150ΔPIX neonatal mice (Sanderson et al., 2012), dissociated with papain, and plated onto 18 mm no. 1.0 coverglass coated with PDL and laminin (BD Biosciences) for imaging (2.4 × 10^6^ cells/12-well). As with rat cells, mouse cells were plated in supplemented MEM, maintained at 37°C and 5% CO_2_, and media was fully exchanged for supplemented Neurobasal-A at 1 DIV. However, mouse cells were fed every 4–5 d and mitotic inhibitors were added at 4–5 DIV.

### Plasmid DNA and cloning

All DNA plasmids, independent of their source, were verified either by Sanger sequencing (University of Colorado Anschutz Barbara Davis Center Sequencing Core Facility) or by whole plasmid sequencing (long-read, Oxford Nanopore Technologies) through Plasmidsaurus prior to their experimental use. Details about specific Sanger sequencing primers are available upon request. Plasmid DNA transformations were carried out in DH5α competent E. coli. The pCAG-hAPP695(Swe-Ind) construct used to drive dendritic spine loss throughout the study was described in (Young-Pearse et al., 2007) (Addgene #30137) and its corresponding pCAG-[ctMCS] empty vector control plasmid (a “pCAGGS”-derived backbone modified by insertion of a Clontech multiple cloning site) was a gift from Dr. Matthew Kennedy, University of Colorado Anschutz Medical Campus. The APP construct codes for full-length human Amyloid-beta precursor protein and harbors the “Swedish” (K595N/M596L) and “Indiana” (V642F) familial Alzheimer's disease (FAD) mutations, driven by the same chimeric “CAG” promoter-enhancer region found in the pCAG-[ctMCS] empty vector. The pCMV-SGFP2-hNFATc3 construct used to initially characterize NFAT-driven spine loss was previously described (Dittmer et al., 2017; Wild et al., 2019) and codes for a well-characterized, C-terminally truncated human NFATc3 fragment (Hoey et al., 1995; Shibasaki et al., 1996; Tomida et al., 2003; Ulrich et al., 2013; Murphy et al., 2014) with an N-terminal SGFP2 tag, driven by the ubiquitous “CMV” promoter.

To determine the role of increased amyloid beta production resulting from the FAD APP mutations in driving dendritic spine loss, the pCAG-hAPP695(M596V) construct was generated by site-directed mutagenesis of pCAG-hAPP695(Swe-Ind) using PCR and Gibson Assembly [New England Biolabs (NEB)]. To do so, a portion of the APP695 CDS located between unique SacI and NotI cut sites was amplified in three segments using long PCR primers (Table 2) such that the resulting fragments shared overlapping sequences at the Swedish and Indiana mutation loci. These overlapping sequences contained the necessary nucleotide substitutions, introduced by primer-target mismatches, to produce the M596V mutation as well as to restore the wild-type K595 and V642 residues. The two outermost fragments also uniquely overlapped with the template sequence immediately upstream of the 5′ SacI cut site and the template sequence immediately downstream of the 3′ NotI cut site. The pCAG-hAPP695(Swe-Ind) backbone was then doubly digested with these same restriction enzymes (SacI-HF and NotI-HF, NEB). The amplified partial inserts and doubly cut vector were all gel purified and combined into a single Gibson Assembly reaction (NEB, Gibson Assembly Master Mix). The mixture contains an exonuclease, a DNA polymerase, a DNA ligase, and suitable buffer. Briefly, in a single reaction, the exonuclease hydrolyzes DNA in the 5′ to 3′ direction to produce complimentary 3′ overhangs from the overlapping end sequences; the overhangs anneal to circularize the plasmid; the polymerase fills any gaps, and the ligase repairs the nicks.

**Table 2.** Gibson PCR primers for APP site-directed mutagenesis

**APP (M596V) Fragment #1**
forward (26 nt): 5′–CGAAACGAAAACCACCGTGGAGCTCC–3′
reverse (27 nt): 5′–GGAATTCTGCATCCAccTTCACTTCAG–3′
**APP (M596V) Fragment #2**
forward (27 nt): 5′–CTGAAGTGAAggTGGATGCAGAATTCC–3′
reverse (31 nt): 5′–CCAAGGTGATcacGATCACTGTCGCTATGAC–3′
**APP (M596V) Fragment #3**
forward (31 nt): 5′–GTCATAGCGACAGTGATCgtgATCACCTTGG–3′
reverse (24 nt): 5′–GAGGGAAAAAGATCTCTCGAGGCG–3′

To explore NFAT-driven spine loss more carefully, the similarly constructed pCMV-SGFP2-mNFATc3 and pCMV-SGFP2-mNFATc4 plasmids were expressly generated for this study. Full-length mouse NFATc3 and full-length mouse NFATc4 were amplified from pCMV-3xHA-mNFATc3 and pCMV-3xHA-mNFATc4 plasmids [presents from Dr. Solange Desagher, Université Montpellier; (Mojsa et al., 2014)], respectively, and subcloned into the pCMV-SGFP2-C1 backbone (Clontech, Takara Bio, catalog #22881). First, NFATc3 was amplified (forward 5′-cgtaagcttatactactgcaaactgtggcgc-3′, reverse 5′-cgatgaattctcactgagcactgtgagaggtcatc-3′) and gel purified to produce an insert with 5′ HindIII and 3′ EcoRI restriction sites. The amplified NFATc3 insert and pCMV-SGFP2-C1 backbone were each digested with HindIII-HF and EcoRI-HF (NEB) in dual restriction digests, gel purified, and ligated with T4 DNA ligase (NEB). In parallel, NFATc4 was similarly amplified (forward 5′-cgactcgaggaggggccgcaagctgcg-3′, reverse 5′-cgatgaattctcaggcaggaggctcttctcc-3′) and purified to produce an insert with 5′ XhoI and 3′ EcoRI restriction sites. The NFATc4 insert and SGFP2-C1 backbone were digested with XhoI and EcoRI-HF (NEB), purified, and ligated with T4 ligase.

Next, the SGFP2-mNFATc3(PSAQAT) mutant was generated by site-directed mutagenesis of pCMV-SGFP2-mNFATc3 using PCR and Gibson Assembly (NEB). To do so, the NFATc3 CDS was amplified in two segments using long PCR primers (Table 3) such that the resulting fragments shared an overlapping sequence that centered around the PxIxIT site and contained the nucleotide substitutions required to produce the PSAQAT mutation, introduced by primer-target mismatches. Additionally, the opposing end of each fragment uniquely overlapped with either the sequence immediately upstream of the 5′ HindIII cut site at the start of the NFATc3 CDS or with the sequence immediately downstream of the 3′ EcoRI cut site at the end of the CDS. The pCMV-SGFP2-C1 backbone was then doubly digested with these same restriction enzymes (HindIII-HF and EcoRI-HF). The amplified partial inserts and doubly cut vector were all gel purified and combined into a single Gibson Assembly reaction (NEB, Gibson Assembly Master Mix).

**Table 3.** Gibson PCR primers for NFATc3 site-directed mutagenesis

**NFATc3 (PSAQAT) Fragment #1**
forward (50 nt): 5′–GTCCGGACTCAGATCTCGAGCTCAAGCTTATACTACTGCAAACTGTGGCG–3′
reverse (60 nt): 5′–GAGAAATGGATGTggcTTGagcACTTGGGCACTCAAAGGGTTTAGGACCACCTAATGGAC–3′
**NFATc3 (PSAQAT) Fragment #2**
forward (60 nt): 5′–CCTTTGAGTGCCCAAGTgctCAAgccACATCCATTTCTCCTAACTGTCATCAAGGAACAG–3′
reverse (45 nt): 5′–CGCGGTACCGTCGACTGCAGAATTCTCACTGAGCACTGTGAGAGG–3′

Lastly, to generate the NFAT knockdown constructs, previously validated murine NFATc3 and NFATc4 mRNA target sequences (Vashishta et al., 2009; Ulrich et al., 2013) were used to design shRNA-coding cassettes. Complimentary pairs of oligos were ordered (Integrated DNA Technologies) (Table 4) such that once annealed, the resulting inserts had either 5′ ApaI and 3′ EcoRI compatible ends for restriction cloning into pmU6-[shRNA] (“pSilencer”), or 5′ HpaI and 3′ XhoI compatible ends for cloning into pmU6-[shRNA]-hSyn-mCh (custom “pFSW” lentiviral shuttle and gift from Dr. Jason Aoto, University of Colorado Anschutz Medical Campus). Accordingly, pmU6-[shRNA] was doubly digested with ApaI and EcoRI-HF while pmU6-[shRNA]-hSyn-mCh was doubly digested with HpaI and XhoI (NEB), the products were gel purified, and compatible backbone-insert pairs were ligated with T4 ligase. The resulting pmU6-NFATc3shRNA and pmU6-NFATc4shRNA constructs were employed by transient transfection, while the pmU6-NFATc3shRNA-hSyn-mCh and pmU6-NFATc4shRNA-hSyn-mCh constructs were used to further produce the knockdown lentiviruses. Irrespective of the transgene delivery method, shRNAs were driven by the same mouse U6 Pol III promoter to ensure similar levels of expression and similar postprocessing by Dicer. However, the constructs intended for lentivirus production also included the neuron-specific human synapsin promoter to drive mCherry expression as a reporter for transduced cells.

**Table 4.** NFATc3 and NFATc4 shRNA cassette oligos

**pmU6-[shRNA]/“pSilencer” Inserts** – ApaI and EcoRI compatible ends	
c3 shRNA cassette, sense oligo 5′–____cATCTTCATTACCTCCATTTTCAAGAGAAATGGAGGTAATGAAGATGTTTTTt____–3′	NFATc3 mRNA target seq. CAUCUUCAUUACCUCCAUU (Vashishta et al., 2009; Ulrich et al., 2013)
c3 shRNA cassette, antisense oligo 3′–ccgggTAGAAGTAATGGAGGTAAAAGTTCTCTTTACCTCCATTACTTCTACAAAAAattaa–5′	
c4 shRNA cassette, sense oligo 5′–____gGGACGGCTCTCCTAGAGATTCAAGAGATCTCTAGGAGAGCCGTCCCTTTTTt____–3′	NFATc4 mRNA target seq. GGGACGGCUCUCCUAGAGA (Vashishta et al., 2009; Ulrich et al., 2013)
c4 shRNA cassette, antisense oligo 3′–ccggcCCTGCCGAGAGGATCTCTAAGTTCTCTAGAGATCCTCTCGGCAGGGAAAAAattaa–5′	
**pmU6-[shRNA]-hSyn-mCh/“pFSW” Inserts** – blunt (HpaI) and XhoI compatible ends	
c3 shRNA cassette, sense oligo 5′–tgCATCTTCATTACCTCCATTTTCAAGAGAAATGGAGGTAATGAAGATGTTTTTTc____–3′	c3 target seq. same as above
c3 shRNA cassette, antisense oligo 3′–acGTAGAAGTAATGGAGGTAAAAGTTCTCTTTACCTCCATTACTTCTACAAAAAAgagct–5′	
c4 shRNA cassette, sense oligo 5′–tgGGGACGGCTCTCCTAGAGATTCAAGAGATCTCTAGGAGAGCCGTCCCTTTTTTc____–3′	c4 target seq. same as above
c4 shRNA cassette, antisense oligo 3′–acCCCTGCCGAGAGGATCTCTAAGTTCTCTAGAGATCCTCTCGGCAGGGAAAAAAgagct–5′	

### Transfection and overexpression

Dissociated hippocampal neurons that were plated on an 18 mm coverglass and maintained in 12-well culture plates for imaging were transfected using Lipofectamine 2000 (Invitrogen) after 12–14 DIV. All transfection conditions for a given experiment received equimolar amounts of plasmid DNA. More specifically, pCAG-[ctMCS] was used as a transfection control for pCAG-hAPP695 constructs, pCMV-SGFP2-C1 was a control for the SGFP2-tagged NFATs, and pmU6-[shRNA]/“pSilencer” was a control for the pmU6-NFATshRNAs. Transfection solutions consisted of 2–3 μl of Lipofectamine and 2–4 μg of plasmid DNA in 150 μl of nonsupplemented Neurobasal-A, per well of a 12-well culture plate (i.e., per coverslip). Solutions were prepared in two parts; a Lipofectamine solution and separate DNA solution were prepared and allowed to sit for 5 min, after which the two were combined and allowed to sit for an additional 20–30 min prior to transfection. One-half of the conditioned media was then removed from each well and reserved, 150 μl of transection solution was applied dropwise, and cells were returned to 37°C and 5% CO_2_. After 75–90 min, the residual media and transfection solution were removed and replaced with the reserved conditioned media and an equal volume of fresh supplemented Neurobasal-A. Neurons were subsequently allowed to express for 48–72 h prior to fixation (48 h for NFAT nuc/cyto; 72 h for spine density).

### Immunocytochemistry and staining

The culture media was removed, and cells were washed with ice-cold ACSF (in mM: 130 NaCl, 5 KCl, 2 CaCl_2_, 1 MgCl_2_, 10 HEPES, 20 glucose), before being fixed for 10 min at room temperature (RT) in a 4% formaldehyde solution in PBS that also included 4% sucrose and 50 mM HEPES (pH 7.4). Fixation solution was then removed, cells were washed three times with PBS, and cells were permeabilized in a 0.1–0.2% Triton X-100 solution in PBS for 10 min at RT on a benchtop rocker. After permeabilization, cells were washed three times and blocked overnight at 4°C in a 4% BSA solution in PBS. The following day, coverslips were incubated for 2 h at RT with rabbit anti-mCh and/or mouse anti-GFP primary antibodies (abcam: ab167453, ab1218 [9F9.F9]) in 4% BSA/PBS. Coverslips were again washed three times and then incubated for 1 h at RT with goat anti-rabbit IgG and/or goat anti-mouse IgG secondary antibodies (Thermo: A-11029, A-11011), conjugated to Alexa 568 and Alexa 488 to amplify detection of mCh and GFP, respectively. Coverslips were washed a final three times and mounted onto glass slides using ProLong Gold (Invitrogen). Where necessary, ProLong Gold with DAPI (Invitrogen) was used to simultaneously stain cell nuclei.

### Epifluorescence microscopy and quantification of spine density and nucleus to cytosol ratio

Imaging was performed on a Zeiss Axiovert 200 M inverted microscope equipped with a Sutter Instruments Lambda LS Xenon Arc Lamp (300 W, 330 nm to 650 nm) or a Sutter Instruments Lambda XL Illuminator (330–700 nm); a Zeiss 63× Plan-Apochromat, 1.4 NA, oil immersion objective; Cy3/Texas Red, FITC/Alexa 488, and DAPI filter sets by Chroma; and a Roper Scientific Photometrics CoolSNAP HQ2 Monochrome CCD camera. Instrumentation and image acquisition were fully controlled by SlideBook 5.5–6.0 software (3i-Intelligent Imaging Innovations). Three-dimensional *z*-stacks were collected over the dendritic arbor with a 200 nm step size, or over the soma with 250 nm steps. *Z*-stacks were then deconvolved using the “nearest-neighbor” algorithm and collapsed into maximum intensity projections of the dendritic arbor or sum intensity projections of the soma.

Subsequent image analysis was performed with Fiji/ImageJ2 software. Spines were counted along 200–300 μm of 2° and 3° apical dendrite. Critically, prior to spine counting, dendrite projection images were pooled across conditions for the given experiment and the image files were blindly renamed using blindanalysis: v1.0/blindrename.pl (Table 1), a Perl script that replaces filenames with pseudorandom alphanumeric strings and creates a .csv keyfile for future unblinding. Dendritic spine counts were thus performed blind, with respect to the underlying overexpression condition, and datasets were unblinded afterward. To quantify the nucleus to cytosol ratio for overexpressed SGFP2-tagged NFATc3 or NFATc4, cells were selected based solely upon morphology as revealed by the mCherry cell fill. Afterward, the DAPI signal and mCherry signal were used to manually generate nuclear and cytosolic masks. The background-subtracted mean fluorescence intensity of the SGFP2 signal within each region was then calculated, and values for the two compartments were compared.

### Lentivirus production

Low passage HEK293FT cells (Invitrogen, catalog #R70007) were cultured at 37°C and 5% CO_2_ in 150 mm dishes using FBS-supplemented DMEM (Gibco). After reaching 60–70% confluence, cells were transfected by calcium phosphate-DNA coprecipitation. Each dish received a 2 ml transfection solution (125 μM CaCl_2_ in HBS) containing 16.25 μg of pCMV-ΔR8.91 packaging plasmid, 8.75 μg of pCMV-VSV-G envelope plasmid, and 25 μg of the relevant pmU6-[shRNA]-hSyn-mCh transfer plasmid (either c3shRNA, c4shRNA, or empty vector control). Additionally, 5 μg of “pEGFP-C1” (or similar) was included as a fill to assess transfection efficiency in the HEK cells. The following day, HEK cell media was exchanged for supplemented Neurobasal-A, and cells were allowed to package and produce lentivirus for 48 h. The culture media was collected, detached HEK cells were pelleted and removed, and the viral supernatant was stored at 4°C until use. Supernatant was added directly to primary hippocampal neuron cultures 5–6 d prior to lysis and sample collection. mCh fluorescence was used to confirm successful infection and to assess efficiency.

### Quantification of mRNA fold-change by qRT-PCR

Culture media was removed, cells were washed with ice-cold ACSF, and RNA was isolated by phenol-chloroform extraction (TRIzol, Invitrogen). Relative transcript abundance was quantified by two-step RT-PCR (Quantitect SYBR Green, Qiagen) using the primers listed in Table 5. Primers for NFATc3 and NFATc4 (Vashishta et al., 2009), and for GAPDH (Garcia et al., 2021) were previously published and validated. Primers for Mdm2 were designed using NCBI Primer-BLAST and validated prior to use. Fold-change was calculated using the 2^−ΔΔCt^ method, commonly referred to as the “delta-delta Ct method”.

**Table 5.** qRT-PCR primers

Primer	
NFATc3 forward: 5′–TGGCATCAACAGTATGGACCTGGA–3′	Vashishta et al., 2009
NFATc3 reverse: 5′–TTTACCACAAGGAGAAGTGGGCCT–3′	
NFATc4 forward: 5′–ATCACTGGCAAGATGGTGGCTACA–3′	Vashishta et al., 2009
NFATc4 reverse: 5′–AGCTTCAGGATTCCAGCACAGTCA–3′	
MDM2 forward: 5′–GATGGCGTAAGTGACCATTCT–3′	NCBI primer-BLAST
MDM2 reverse: 5′–GCAGGGCTTATTCCTCTTCTT–3′	
GAPDH forward: 5′–GATGCTGGTGCTGAGTATGT–3′	Garcia et al., 2021
GAPDH reverse: 5′–GCTGACAATCTTGAGGGAGTT–3′	

### Quantification of protein fold-change by immunoblotting

Culture media was removed, and cells were washed with ice-cold ACSF just prior to cell lysis and protein solubilization by RIPA buffer (150 mM NaCl, 50 mM Tris pH 8.0, 1% IGEPAL/NP-40, 1% deoxycholate, 0.1% SDS, 1 mM EDTA pH 8.0, 10 mM NaF, 2 μg/ml pepstatin and leupeptin, protease inhibitor cocktail—Roche cOmplete). Protein concentrations were determined by BCA (Thermo Pierce). Lysates were sonicated and then boiled in sample buffer with 1% SDS and 2.5% BME. Protein was resolved by Tris glycine SDS-PAGE, transferred to PVDF membrane, and blocked overnight at 4°C in 3% BSA in TBS-T. Blots were incubated for 2 h at RT in mouse anti-MDM2 (Santa Cruz, sc-965), mouse anti-actin (Millipore, MAB1501), mouse anti-GAPDH (GeneTex, GTX627408), or mouse anti-PSD95 (BioLegend, 810401) primary antibodies in 1.5% BSA/TBS-T. Blots were washed three times in TBS-T, and incubated for 1 h at RT in HRP-conjugated goat anti-mouse IgG (H + L) secondary antibody (Bio-Rad). Immunoreactivity was detected by ECL (SuperSignal West Pico or West Femto Substrate) using an Alpha Innotech AlphaImager.

### Quantification of ubiquitination fold-change by immunoprecipitation and immunoblotting

As above, culture media was removed, and cells were washed with ice-cold ACSF just prior to cell lysis and protein solubilization by RIPA buffer. Approximately 5% of each lysate was reserved for input and prepared with sample buffer under reducing conditions. The remainder of each lysate was then split into a pair of samples, and each was then brought up to volume with IP buffer (150 mM NaCl, 20 mM Tris pH 7.4, 5 mM EDTA, 0.5% Triton) and either PSD95 (BioLegend, 810401) or ubiquitin (Santa Cruz, sc-8017) antibodies. Protein A agarose beads were pre-equilibrated with IP buffer, added to each sample, and samples were tumbled for 1.5 h at 4°C. Beads were then washed three times in IP buffer and added to sample buffer containing DTT. Beads were then boiled in sample buffer with DTT for 5 min prior to SDS-PAGE and immunoblotting as described above.

### Soluble Aβo preparation

Lyophilized Aβ1–42 (AnaSpec) was first fully dissolved in HFIP at RT. Aliquots were allowed to dry and Aβ was stored as a dried film at −80°C. To prepare Aβo, the peptide film was first dissolved in anhydrous DMSO to a concentration of 5 mM, then diluted to 100 μM with PBS, vortexed and allowed to oligomerize overnight at 4°C. The preparation was centrifuged at 14,000 × *g* for 10 min at 4°C to remove insoluble aggregates. The supernatant was kept on ice until use (20–30 min) and was applied to cells at a final concentration of 500 nM. This protocol was reliably shown to produce an Aβ sample enriched for soluble, low molecular weight (dimer/trimer/tetramer) Aβo in previous studies (Freund et al., 2016; Actor-Engel et al., 2021).

### Statistical analysis

All statistical analyses were performed using GraphPad Prism 6–8. Statistical significance for comparisons between two groups was determined by two-tailed unpaired *t* tests, with Welch's correction for unequal variances where appropriate. For comparisons between more than two groups, significance was determined by ordinary one-way ANOVA with various post hoc corrections for family-wise type I error; Sidak's, Tukey's, or Dunnett's correction was used as appropriate. Where individual groups were compared to a normalized theoretical mean of 1 (e.g., mRNA and protein fold-change), a one-sample *t* test was used. And when multiple groups were compared to a normalized theoretical mean of 1, Bonferroni's correction for multiple comparisons was applied.

## Results

### Overexpression of an APP FAD mutant that overproduces Aβ requires NMDAR conformational signaling and Ca^2+^ influx through CP-AMPARs to drive dendritic spine loss

**Figure 1.**
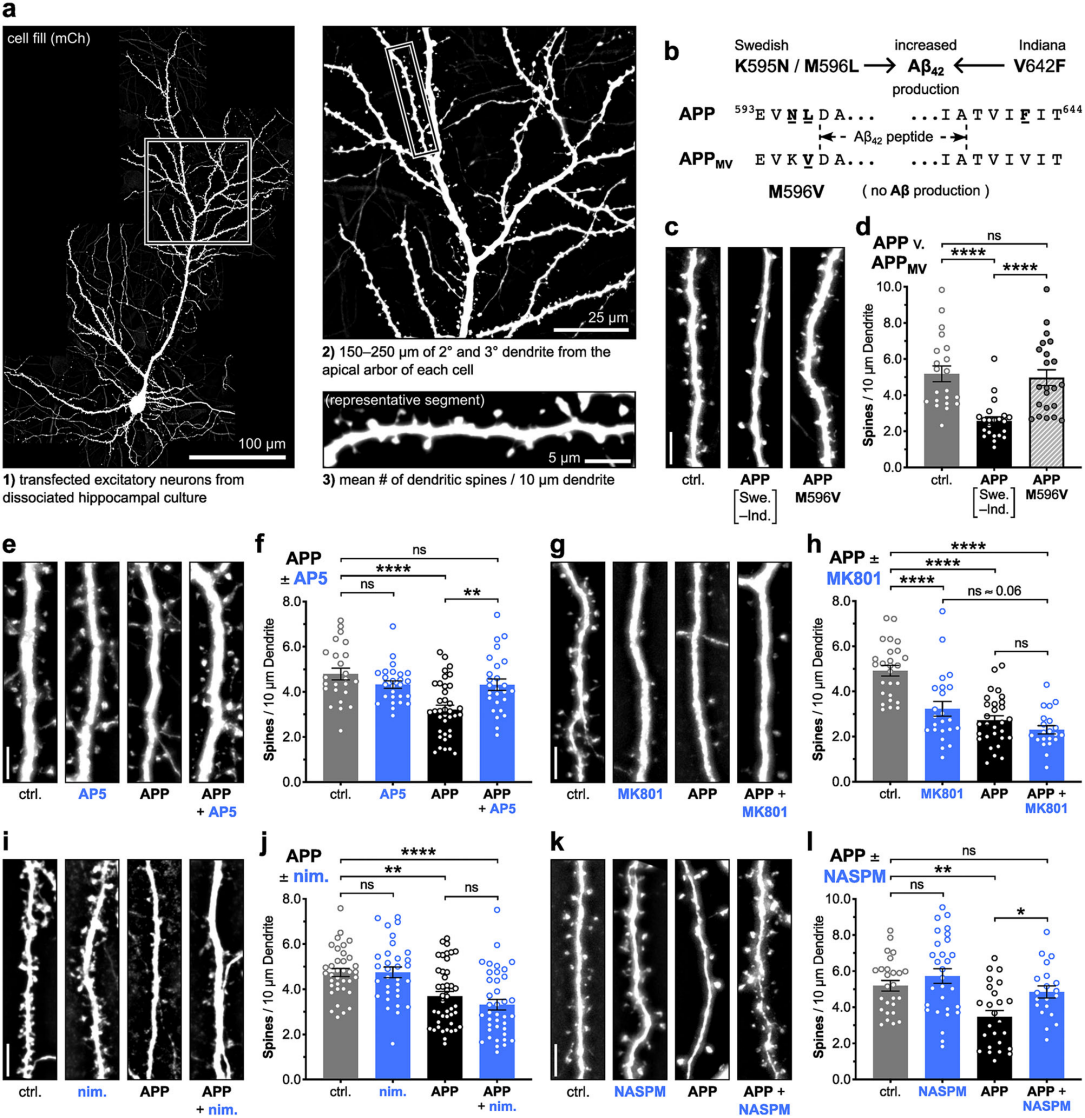
APP overexpression-mediated Aβ production requires NMDARs and CP-AMPARs, but not LTCCs, to drive dendritic spine loss in hippocampal neurons. ***a***, Representative cultured hippocampal neuron, and spine density quantification workflow. ***b***, Amino acid sequences for APP and APP_MV_ with their respective mutations shown in relation to the Aβ peptide sequence embedded within APP695. ***c*,*e*,*g*,*i*,*k***, Representative images of dendrite from dissociated rat hippocampal neurons expressing mCherry (mCh) as cell fill (5 μm scale bar). Cells were co-transfected at DIV 12–14 with mCh and either control plasmid, FAD-mutant APP plasmid, or APP_MV_ plasmid, allowed to overexpress for 72 h with or without bath-applied drug/vehicle, and fixed at DIV 15–17. Drug or vehicle was bath-applied following transfection and remained present for the duration of the overexpression period. ***d***, Quantification of mean dendritic spines per 10 μm dendrite (±SEM) for control, APP, and APP_MV_ overexpression conditions. *n* = 21–22 cells per condition, *N* = 3 cultures. ****p < 0.0001 by one-way ANOVA with Tukey's correction. ***f*,*h*,*j*,*l***, Quantification of mean dendritic spines per 10 μm dendrite (±SEM) for control and APP overexpression conditions, in combination with drug or vehicle. ***f***, 100 μM D,L-AP5 (in H_2_O), *n* = 24–37 cells per condition, *N* = 4 cultures. ***h***, 10 μM MK801 (in H_2_O), *n* = 21–29 cells per condition, *N* = 3 cultures. ***j***, 5 μM nimodipine (in DMSO), *n* = 32–43 cells per condition, *N* = 3 cultures. ***l***, 20 μM NASPM (in H_2_O), *n* = 20–29 cells per condition, *N* = 3 cultures. *p < 0.05, **p < 0.01, and ****p < 0.0001 by one-way ANOVA with Sidak's correction.

To further interrogate postsynaptic CaN signaling pathways required for Aβ-induced dendritic spine elimination, we overexpressed an APP construct harboring Swedish (K595N/M596L) and Indiana (V642F) FAD mutations that strongly promote Aβ_42_ overproduction (referred to here as simply APP) (Young-Pearse et al., 2007) to drive spine loss in cultured hippocampal neurons (****p < 0.0001 for APP to ctrl., one-way ANOVA with Tukey's; Fig. 1*a–d*). To confirm that the observed spine loss resulting from FAD mutant APP overexpression was indeed due specifically to the overproduction of Aβ, we also tested the overexpression of an APP construct harboring the M596V mutation (Fig. 1*b*, referred to as APP_MV_) that prevents β-secretase cleavage and all subsequent Aβ production (Citron et al., 1995; Kamenetz et al., 2003; Hsieh et al., 2006). Accordingly, we found that overexpression of the APP_MV_ construct did not drive dendritic spine loss when compared to control and APP overexpression (n.s. for APP_MV_ to ctrl., ****p < 0.0001 for APP_MV_ to APP, one-way ANOVA with Tukey's; Fig. 1*c,d*).

Moving forward with the use of FAD mutant APP overexpression, we then employed an array of pharmacologic and genetic manipulations to identify signaling events required for subsequent spine loss. First, we confirmed NMDAR involvement using the competitive antagonist AP5 and further characterized this receptor's role using the uncompetitive antagonist and use-dependent pore blocker MK801. Application of 100 μM D,L-AP5 (50 μM D-AP5), which competes with glutamate binding to GluN2 subunits, during the entire 72 h period of APP overexpression prevented spine loss and preserved normal spine density in dissociated rat hippocampal neurons (**p < 0.01 for APP + AP5 to APP, n.s. for APP + AP5 to ctrl., one-way ANOVA with Sidak's; Fig. 1*e,f*). Interestingly, 10 μM (+)-MK-801, which blocks Ca^2+^ influx through the channel pore, not only failed to block APP overexpression-induced spine loss as seen in some previous studies (Kessels et al., 2013; Birnbaum et al., 2015), but also significantly reduced dendritic spine density when applied on its own for 72 h (n.s. for APP + MK801 to APP, ****p < 0.0001 for APP + MK801 to ctrl., ****p < 0.0001 for MK801 to ctrl., one-way ANOVA with Sidak's; Fig. 1*g,h*). The ability of MK801 to independently drive spine loss, although not previously reported, is consistent with studies indicating that glutamate-dependent, nonionotropic NMDAR signaling, isolated by either MK801 or GluN1 glycine-site antagonists (e.g., 7CK), is sufficient to drive LTD and spine shrinkage (Nabavi et al., 2013; Stein et al., 2015), which in turn can promote spine elimination. However, the impact of MK801 alone on spine density also raises the question of whether Aβ solely engages nonionotropic NMDAR signaling to drive spine loss as some previous studies might suggest (Kessels et al., 2013; Birnbaum et al., 2015), or if Aβ also engages other Ca^2+^ sources that couple to downstream Ca^2+^-dependent pathways, including those reliant on CaN.

To this end, we next used the dihydropyridine blocker nimodipine and the uncompetitive antagonist and use-dependent pore blocker NASPM, to test for L-type voltage-gated Ca^2+^ channel (LTCC) and CP-AMPAR involvement, respectively. Although 5 μM nimodipine failed to block APP/Aβ-induced spine loss (n.s. for APP + nim. to APP, ****p < 0.0001 for APP + nim. to ctrl., one-way ANOVA with Sidak's; Fig. 1*i,j*), 20 μM NASPM did successfully block it, preserving normal spine density (*p < 0.05 for APP + NASPM to APP, n.s. for APP + NASPM to ctrl., one-way ANOVA with Sidak's; Fig. 1*k,l*). Importantly, previous studies found that Aβ can rapidly recruit CP-AMPARs to the synapse in hippocampal neurons (Whitcomb et al., 2015; Sanderson et al., 2021) and that their synaptic incorporation is required for Aβ-induced inhibition of LTP (Sanderson et al., 2021). Thus, Aβ-mediated spine loss may require both ionotropic, Ca^2+^-dependent CP-AMPAR signaling and nonionotropic, Ca^2+^-independent conformational NMDAR signaling.

### APP/Aβ-induced spine loss requires AKAP150-CaN anchoring, in addition to CaN phosphatase activity

**Figure 2.**
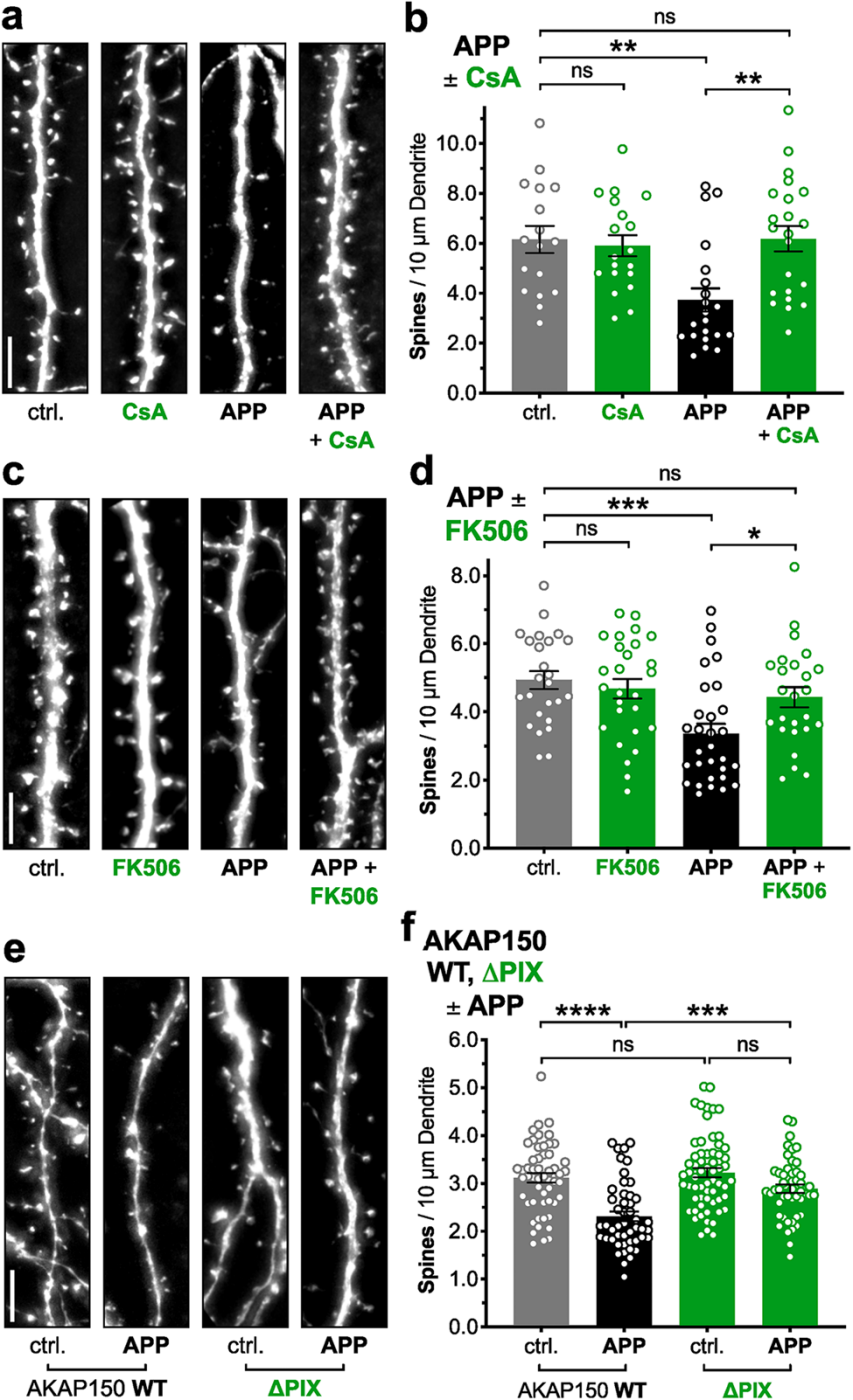
APP overexpression-induced spine loss requires AKAP150-CaN anchoring, in addition to CaN phosphatase activity. ***a*,*c***, Representative dendrite images from dissociated rat hippocampal neurons expressing mCh (5 μm scale bar). Cells were co-transfected at DIV 12–14 with mCh and either control or APP plasmids, allowed to overexpress for 72 h with or without drug/vehicle, and fixed at DIV 15–17. ***b*,*d***, Mean dendritic spines/10 μm dendrite (±SEM) for ctrl. and APP overexpression conditions, with either drug or vehicle. ***b***, 2 μM CsA (in DMSO), *n* = 17–22 cells per condition, *N* = 3 cultures. ***d***, 5 μM FK506 (in DMSO), *n* = 25–30 cells per condition, *N* = 3 cultures. ***e***, Representative dendrite images from dissociated WT or AKAP150ΔPIX mouse hippocampal neurons expressing EGFP (5 μm scale bar); ΔPIX is a KI deletion of AKAP150's CaN-docking PxIxIT motif. Cells were co-transfected at DIV 12 with EGFP and either control or APP plasmids, allowed to overexpress for 48–72 h, and fixed at DIV 14–15. ***f***, Mean dendritic spines/10 μm dendrite (±SEM) for ctrl. and APP overexpression conditions, on WT and ΔPIX backgrounds. *n* = 48–62 cells per condition, *N* = 6 cultures. *p < 0.05, **p < 0.01, ***p < 0.001, and ****p < 0.0001 by one-way ANOVA with Sidak's correction.

We then confirmed the previously established downstream involvement of the catalytic activity of the Ca^2+^/CaM-dependent phosphatase CaN in APP/Aβ-mediated spine loss using the immunosuppressant CaN inhibitors CsA and FK506 (Hsieh et al., 2006; Shankar et al., 2007). As expected, 2 μM CsA blocked 72 h APP overexpression-induced spine loss and preserved normal spine density in dissociated rat hippocampal neurons (**p < 0.01 for APP + CsA to APP, n.s. for APP + CsA to ctrl., one-way ANOVA with Sidak's; Fig. 2*a,b*), as did 5 μM FK506 (*p < 0.05 for APP + FK506 to APP, n.s. for APP + FK506 to ctrl., one-way ANOVA with Sidak's; Fig. 2*c,d*). However, we further examined a potential role for local, postsynaptic CaN signaling that depends on anchoring to the scaffold protein AKAP150. Importantly, genetic disruption of postsynaptic CaN anchoring to AKAP150, through deletion of the CaN-docking PxIxIT motif in AKAP150ΔPIX knock-in mice, prevents AMPAR dephosphorylation and removal from synapses during LTD (Sanderson et al., 2012, 2016) and also protects against acute inhibition of LTP by Aβ (Sanderson et al., 2021). Accordingly, 48–72 h APP overexpression in WT mouse hippocampal cultures induced robust spine loss, while APP overexpression in hippocampal cultures from AKAP150ΔPIX mice failed to significantly impact spine density (***p < 0.001 for APP on ΔPIX background to APP on WT background, n.s. for APP to ctrl. on ΔPIX background, one-way ANOVA with Sidak's; Fig. 2*e,f*).

### APP/Aβ-induced spine loss requires NFAT transcription factors

**Figure 3.**
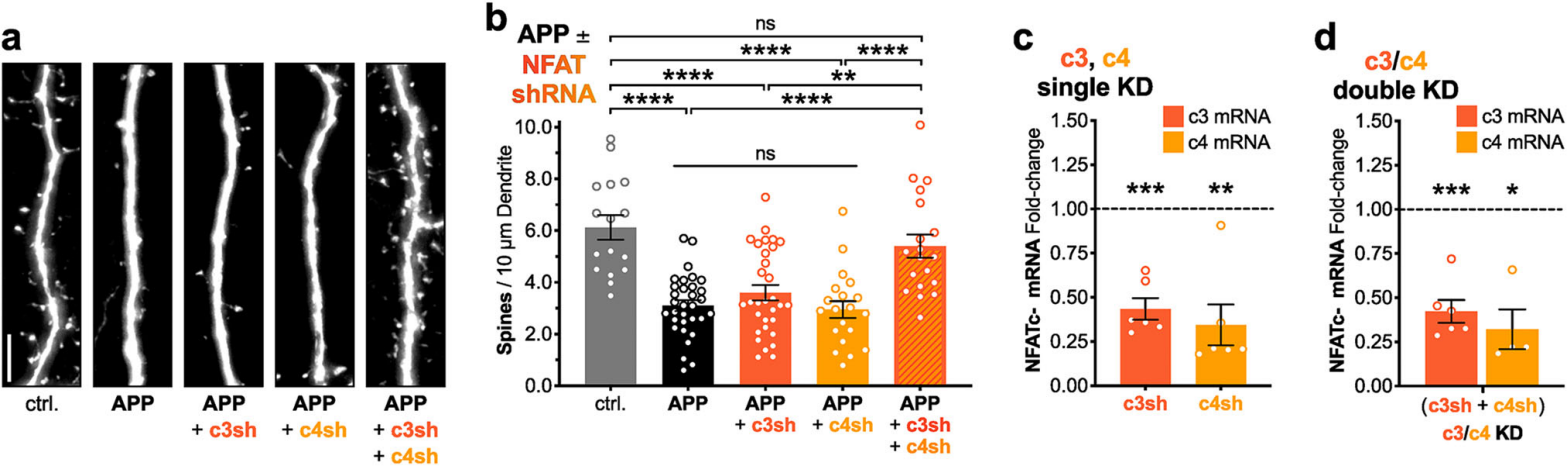
APP overexpression-induced spine loss requires NFAT transcription factors. ***a***, Representative dendrite images from dissociated rat hippocampal neurons expressing mCh (5 μm scale bar). Cells were co-transfected at DIV 12–14 with mCh and either control plasmid, APP plasmid, or a combination of APP plasmid and plasmid(s) encoding NFAT shRNA. Cells were allowed to overexpress for 72 h and fixed at DIV 15–17. ***b***, Mean dendritic spines/10 μm dendrite (±SEM) for ctrl., APP, and APP + shRNA overexpression conditions. *n* = 16–32 cells per condition, *N* = 3 cultures. **p < 0.01 and ****p < 0.0001 by one-way ANOVA with Sidak's correction. ***c,d***, Mean fold-change (±SEM) in NFAT mRNA by RT-qPCR for rat hippocampal cultures infected by one or more NFAT shRNA lentiviruses, relative to cultures infected with corresponding amounts of control virus. Cultures were infected at DIV 10–11 and RNA was harvested at DIV 15–16. ***c***, single KD, *n* = 6 samples each, from *N* = 3 cultures. **p < 0.005, ***p < 0.0005 by one-sample *t* test with theoretical mean of 1.0 and Bonferroni's correction (*p *< *α*/2, *α *= 0.05). ***d***, double KD, *n* = 6 samples, *N* = 3 cultures. *p < 0.01$\bar{6}$, ***p < 0.000$\bar{3}$ by one-sample *t* test with theoretical mean of 1.0 and Bonferroni's correction (*p *< *α*/3, *α* = 0.05). Double KD samples and their controls were later rerun to quantify MDM2 mRNA fold-change in response to NFATc3/c4 double KD and quantified in Figure 6*d*.

Previous studies implicated NFAT-mediated transcriptional signaling downstream of CaN in Aβ-induced dendritic spine loss (Wu et al., 2010; Hudry et al., 2012). However, the approaches employed to disrupt CaN-NFAT interactions in these studies also disrupt CaN interactions with many other signaling proteins and substrates (Li et al., 2011; Goldman et al., 2014; Gibson et al., 2019), including the AKAP150 scaffold (Dell'Acqua et al., 2002; Oliveria et al., 2007; Li et al., 2012; Murphy et al., 2014) and other transcription factors, such as CRTC1, which has been implicated in LTD and Aβ-induced neuronal dysfunction (Espana et al., 2010; Nonaka et al., 2014; Pan et al., 2021). Furthermore, although previous studies implicated a potential role for NFATc4 in Aβ-mediated spine loss, a role for the other predominant isoform in the hippocampus, NFATc3, was not investigated. Thus, to characterize the necessity of individual NFATc family members in Aβ-induced dendritic spine elimination (Fig. 3*a,b*), we generated shRNA constructs using previously validated short hairpin sequences targeting all known and predicted mouse and rat mRNA variants of NFATc3 and NFATc4 (Vashishta et al., 2009; Ulrich et al., 2013). We validated knockdown efficiency of these shRNAs by lentiviral transduction, followed by 5–6 d of expression and then RT-qPCR. In rat hippocampal neurons, individual expression of NFATc3 and NFATc4 shRNAs significantly knocked down the relative abundance of their target mRNAs by ∼57% and ∼66%, respectively (***p < 0.0005 for c3 mRNA, **p < 0.005 for c4 mRNA, one-sample *t* test, theoretical mean 1.0, Bonferroni's *p *< *α*/2 and *α* = 0.05; Fig. 3*c*). Similarly, viral co-transduction and subsequent co-expression of these NFATc3 and NFATc4 shRNAs significantly knocked down the relative abundance of both target mRNAs by ∼58% and ∼68%, respectively (***p < 0.000$\bar{3}$ for c3 mRNA, *p < 0.01$\bar{6}$ for c4 mRNA, one-sample *t* test, theoretical mean 1.0, Bonferroni's *p *< *α*/3 and *α* = 0.05; Fig. 3*d*). Next, we tested the effects of single and double NFAT isoform knockdowns on APP overexpression-induced spine loss by co-transfection of one or more NFAT shRNAs alongside 72 h APP overexpression. Single isoform knockdowns of NFATc3 and NFATc4 each failed to protect against APP overexpression-induced spine loss (****p < 0.0001 for APP + c3sh to ctrl. and for APP + c4sh to ctrl., n.s. for APP + c3sh to APP and for APP + c4sh to APP, one-way ANOVA with Sidak's; Fig. 3*a,b*). Importantly, however, double knockdown of NFATc3 and NFATc4 isoforms successfully blocked APP overexpression-induced spine loss and preserved normal spine density (****p < 0.0001 for APP + c3sh + c4sh to APP, n.s. for APP + c3sh + c4sh to ctrl., one-way ANOVA with Sidak's; Fig. 3*a,b*).

### Overexpression of NFATc3, but not NFATc4, recapitulates APP/Aβ-induced spine loss

**Figure 4.**
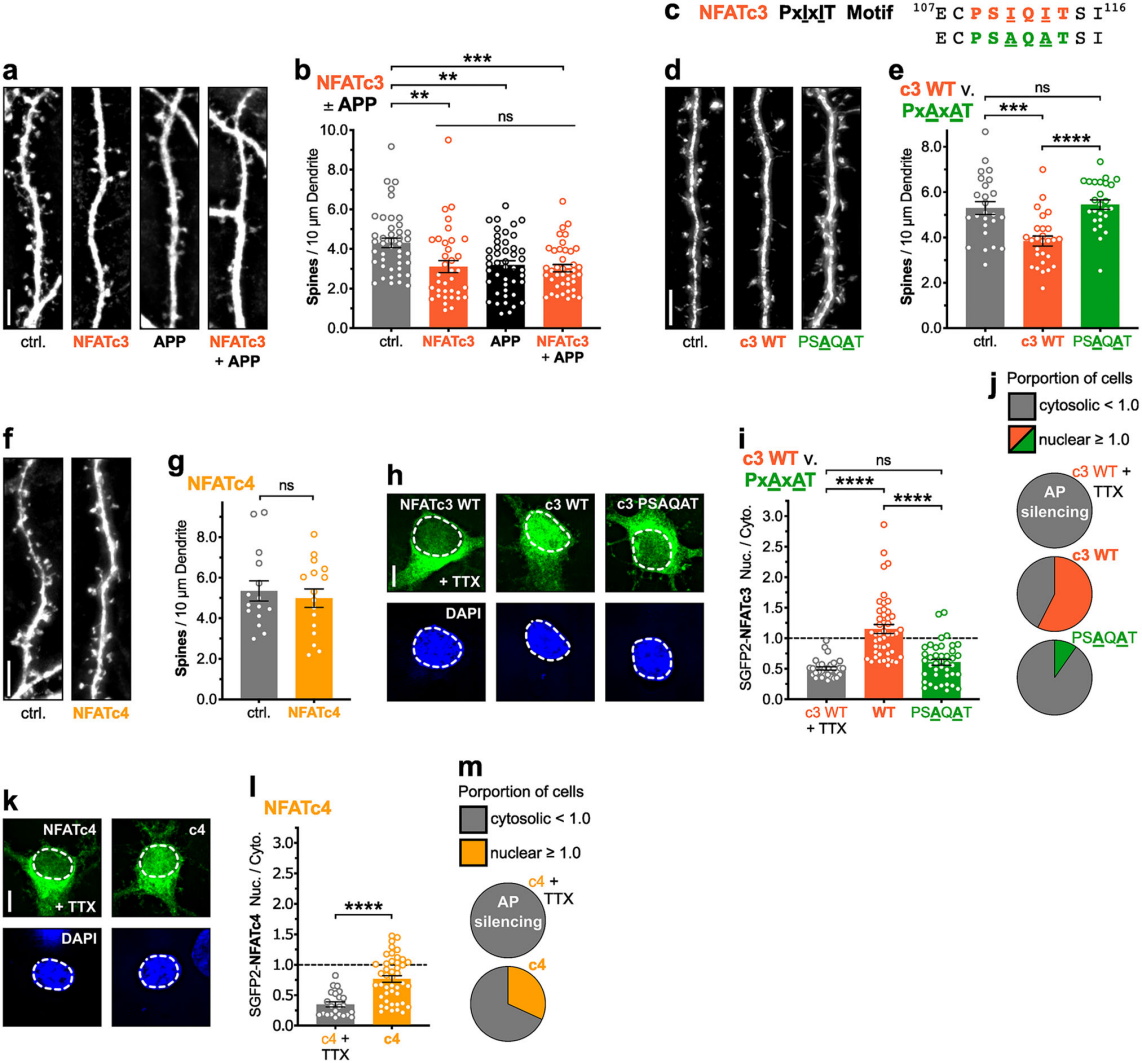
Overexpression of NFATc3, but not NFATc4, recapitulates APP overexpression-induced spine loss. ***a,d,f***, Representative dendrite images from dissociated rat hippocampal neurons expressing mCh (5 μm scale bar). Cells were co-transfected at DIV 12–14 with either control plasmid(s), NFAT plasmid, APP plasmid, or a combination of these plasmids, allowed to express for 72 h, and fixed at DIV 15–17. ***b***, Mean dendritic spines/10 μm dendrite (±SEM) for ctrl., NFATc3, APP, and APP + NFATc3 overexpression conditions. *n* = 36–45 cells per condition, *N* = 3 cultures. **p < 0.01, ***p < 0.001 by one-way ANOVA with Sidak's correction. ***c***, CaN-binding PxIxIT site amino acid sequences for NFATc3 WT (PSIQIT) and alanine-substituted PSAQAT mutant; loss of conserved isoleucines greatly diminishes CaN binding affinity. ***e,g***, Mean dendritic spines/10 μm dendrite (±SEM) for ctrl., NFATc3 WT, NFATc3 PSAQAT, and NFATc4 overexpression conditions. ***e***, NFATc3, *n* = 25–27 cells per condition, *N* = 3 cultures. ***p < 0.001, and ****p < 0.0001 by one-way ANOVA with Tukey's correction. ***g***, NFATc4, *n* = 15–16 cells per condition, *N* = 3 cultures. ***h*,*k***, Representative images of the soma from dissociated rat hippocampal neurons expressing SGFP2-tagged NFATc3 WT, NFATc3 PSAQAT, or NFATc4, in the presence of spontaneous activity or bath-applied TTX (5 μm scale bar). ROI indicates nuclear mask determined by DAPI staining. Cells were co-transfected at DIV 12–13 with mCh (cell fill) and an SGFP2-NFAT and then fixed 48 h later at DIV 14–15. Where indicated, 1 μM TTX was applied for 2 h prior to fixation. ***i,l***, Quantification of mean nucleus to cytosol ratio (±SEM) for SGFP2-NFAT fluorescence in spontaneously active and TTX-silenced neurons. ***i***, NFATc3, *n* = 31–47 cells per condition, *N* = 3 cultures. ****p < 0.0001 by one-way ANOVA with Tukey's correction. ***l***, NFATc4, *n* = 23–44 cells per condition, *N* = 3 cultures. ****p < 0.0001 by two-tailed, unpaired *t* test with Welch's correction for unequal variances. ***j*,*m***, The proportion of cells where NFAT localized predominantly to the nucleus (nuc./cyto. ≥ 1) and the proportion where NFAT localized predominantly to the cytosol (nuc./cyto. < 1), for each condition quantified in panels ***i*** and ***l***.

To test the sufficiency of NFATc isoforms in driving dendritic spine loss in the absence of upstream Aβ-dependent signaling, we characterized the impacts of overexpression of NFATc constructs in rat hippocampal neurons (Fig. 4*a–g*). First, we overexpressed NFATc3 for 72 h with or without co-overexpression of APP (Fig. 4*a,b*). Remarkably, 72 h overexpression of NFATc3 drove the same degree of dendritic spine loss as APP overexpression, and interestingly their co-overexpression failed to produce a greater degree of cumulative spine loss (**p < 0.01 for NFATc3 to ctrl., n.s. for NFATc3 to APP and for NFATc3 + APP to NFATc3, one-way ANOVA with Sidak's; Fig. 4*a,b*). Next, to explore NFATc3 overexpression-induced dendritic spine loss using a loss-of-function approach, we generated a CaN binding-deficient NFATc3 mutant (PSAQAT) (Li et al., 2011) by substituting alanine for the conserved isoleucine residues of the CaN-binding PxIxIT motif (Fig. 4*c*). As in Figure 4*a,b*, 72 h overexpression of NFATc3 (WT) drove significant dendritic spine loss (***p < 0.001 for c3 WT to ctrl., one-way ANOVA with Tukey's; Fig. 4*d,e*). Critically, however, PxAxAT mutation of the PxIxIT motif blocked this 72 h overexpression effect of NFATc3, preserving normal spine density (****p < 0.0001 for c3 PSAQAT to c3 WT, n.s. for c3 PSAQAT to ctrl., one-way ANOVA with Tukey's; Fig. 4*d,e*). Similarly, in parallel experiments, we overexpressed NFATc4 for 72 h, but failed to observe significant dendritic spine loss (n.s. for NFATc4 to ctrl., unpaired two-tailed *t* test; Fig. 4*f,g*).

To then better understand the results of Figure 4*a–g* in the context of the differing degrees of activity-responsiveness previously reported for these two NFATc isoforms (Ulrich et al., 2013; Wild et al., 2019), we overexpressed each of the NFATc3 and NFATc4 constructs from Figure 4*c–g* in rat hippocampal neurons and characterized their nuclear enrichment in response to spontaneous activity (Fig. 4*h–m*). Notably, NFATc3 WT was enriched within the nucleus (nuc./cyto ≥ 1.0) in a majority of cells in the presence of spontaneous activity, while the PSAQAT mutation relegated NFATc3 to the cytosol (nuc./cyto < 1.0) to a similar degree as NFATc3 WT in the presence of 1 μM TTX to silence neuronal activity by blocking action potential firing (****p < 0.0001 for c3 PSAQAT to WT, n.s. for c3 PSAQAT to WT + TTX, one-way ANOVA with Tukey's; Fig. 4*h–j*). Additionally, NFATc4 remained largely cytosolic in a majority of cells, despite the presence of spontaneous activity (Fig. 4*k–m*). These findings, combined with those of the RNAi experiments, indicate that, while NFATc3 and NFATc4 may both participate in driving Aβ-mediated spine loss, NFATc3 may play a much more prominent role than previously appreciated.

### Soluble Aβo increase NFATc3 nuclear localization via NMDAR and CP-AMPAR signaling, while NFATc4 is unresponsive

**Figure 5.**
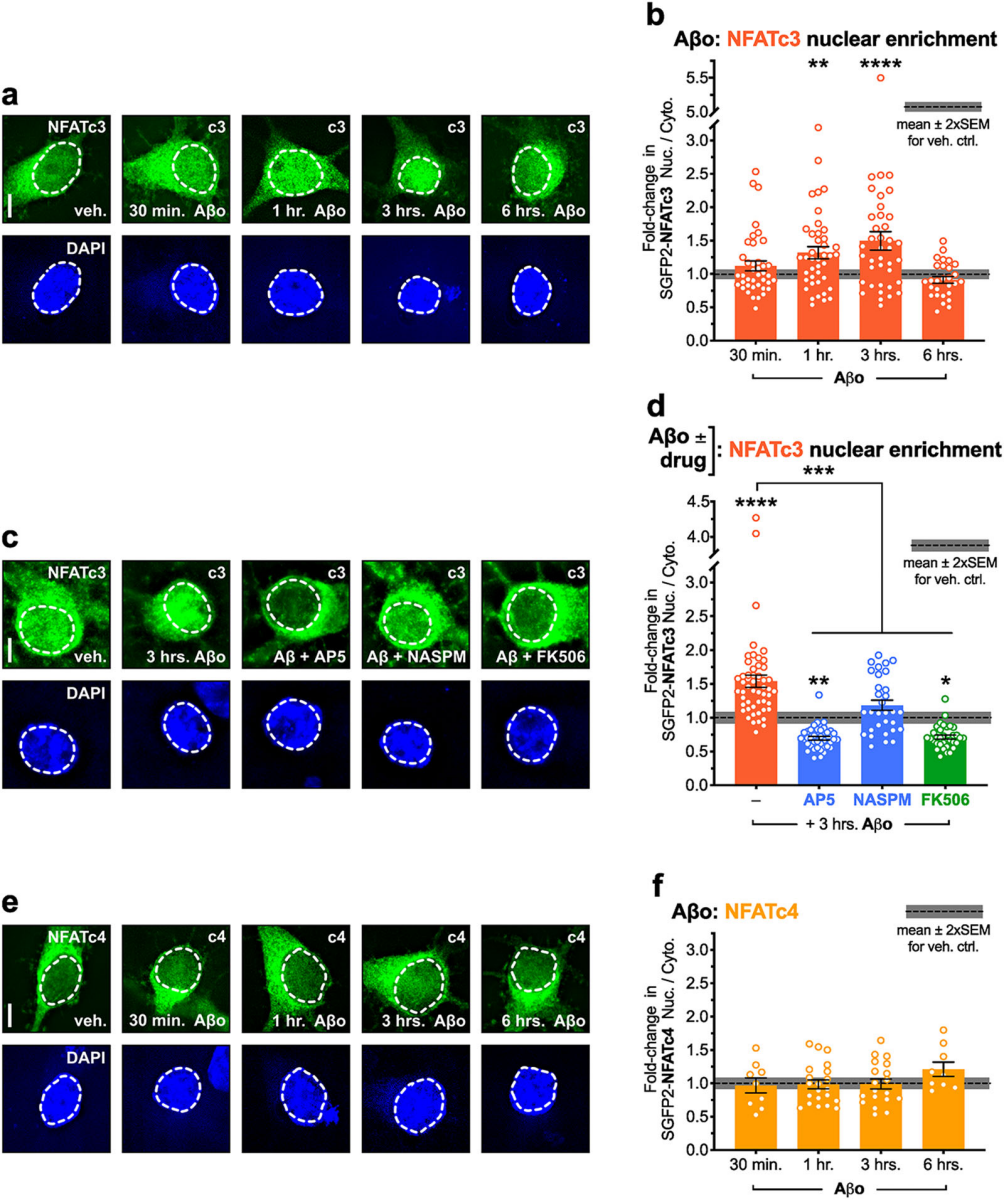
Soluble Aβ oligomers (Aβo) increase NFATc3 nuclear localization via NMDAR and CP-AMPAR signaling, while NFATc4 appears unresponsive. ***a*,*c*,*e***, Representative soma images from dissociated rat hippocampal neurons expressing SGFP2-tagged NFATc3 or NFATc4, in the presence of spontaneous activity and bath-applied Aβo or vehicle (PBS/DMSO), with or without additional drugs (AP5, NASPM, or FK506) as indicated (5 μm scale bar). Cells were co-transfected at DIV 12–13 with mCh (cell fill) and an SGFP2-NFAT. 48 h later (DIV 14–15), cells were treated with 500 nM soluble Aβo for the indicated amount of time prior to fixation. ***b*,*d*,*f***, Quantification of mean fold-change (±SEM) in nucleus to cytosol ratio for SGFP2-NFAT fluorescence in Aβo-treated cells, relative to vehicle-treated control cells. ***b***, NFATc3, *n* = 28–41 cells per time point, *N* = 3 cultures. ***d***, NFATc3 (Aβo ± drug), *n* = 31–51 cells per condition, *N* = 3 cultures. ***f***, NFATc4, *n* = 9–21 cells per time point, *N* = 3 cultures. *p < 0.05, **p < 0.01, ***p < 0.001, ****p < 0.0001 by one-way ANOVA with Dunnett's correction (***b,f***) or Sidak's correction (***d***).

Following up on these results, we next characterized the fold-change in NFATc nuclear enrichment or lack thereof, in response to acute exposure to bath-applied 500 nM soluble Aβo. Interestingly, NFATc3 displayed significant 1.3- and 1.5-fold increases in its nuclear enrichment following 1 and 3 h exposures to Aβo, respectively (**p < 0.01 for 1 h and ****p < 0.0001 for 3 h, one-way ANOVA with Dunnett's; Fig. 5*a,b*), We repeated this experiment using 3 h Aβo exposure, either alone or in the presence of one of several pharmacologic inhibitors that were previously shown to block APP/Aβ-induced spine loss in Figures 1 and 2. As anticipated, concurrent bath application of either 100 μM D,L-AP5 (50 μM D-AP5), 20 μM NASPM, or 5 μM FK506 blocked NFATc3 nuclear enrichment following 3 h of exposure to 500 nM Aβo (***p < 0.001 for AP5, NASPM, and FK506 to Aβo alone, one-way ANOVA with Sidak's; Fig. 5*c,d*). However, while both AP5 and FK506 blocked Aβo-induced nuclear enrichment and significantly reduced overall nuclear occupancy below the level observed under control conditions with spontaneous activity alone (**p < 0.01 for AP5 to veh. ctrl., *p < 0.05 for FK506, one-way ANOVA with Sidak's; Fig. 5*c,d*), NASPM only blocked Aβo-induced enrichment, effectively restoring NFATc3 nuclear occupancy to control levels in the presence of spontaneous activity alone (n.s. for NASPM to veh. ctrl., one-way ANOVA with Sidak's; Fig. 5*c,d*). These results indicate that NMDARs and CaN are required for NFATc3 regulation both under control conditions and in the presence of Aβ, consistent with their well-established roles in mediating NFAT activation in response excitatory synaptic input (Graef et al., 1999; Wild et al., 2019). However, these results also suggest that Aβ specifically engages CP-AMPAR signaling to enhance NFATc3 activation. In contrast, no change was observed in NFATc4 localization following any length of Aβo exposure (n.s. for all time points, one-way ANOVA with Dunnett's; Fig. 5*e,f*). These findings further indicate that NFATc3 may play a more important and even a dominant role in neuronal responses to Aβ than previously thought.

### APP/Aβ-induced spine loss requires the E3-ubiquitin ligase Mdm2, and Aβ-induced Mdm2 upregulation requires NFAT

**Figure 6.**
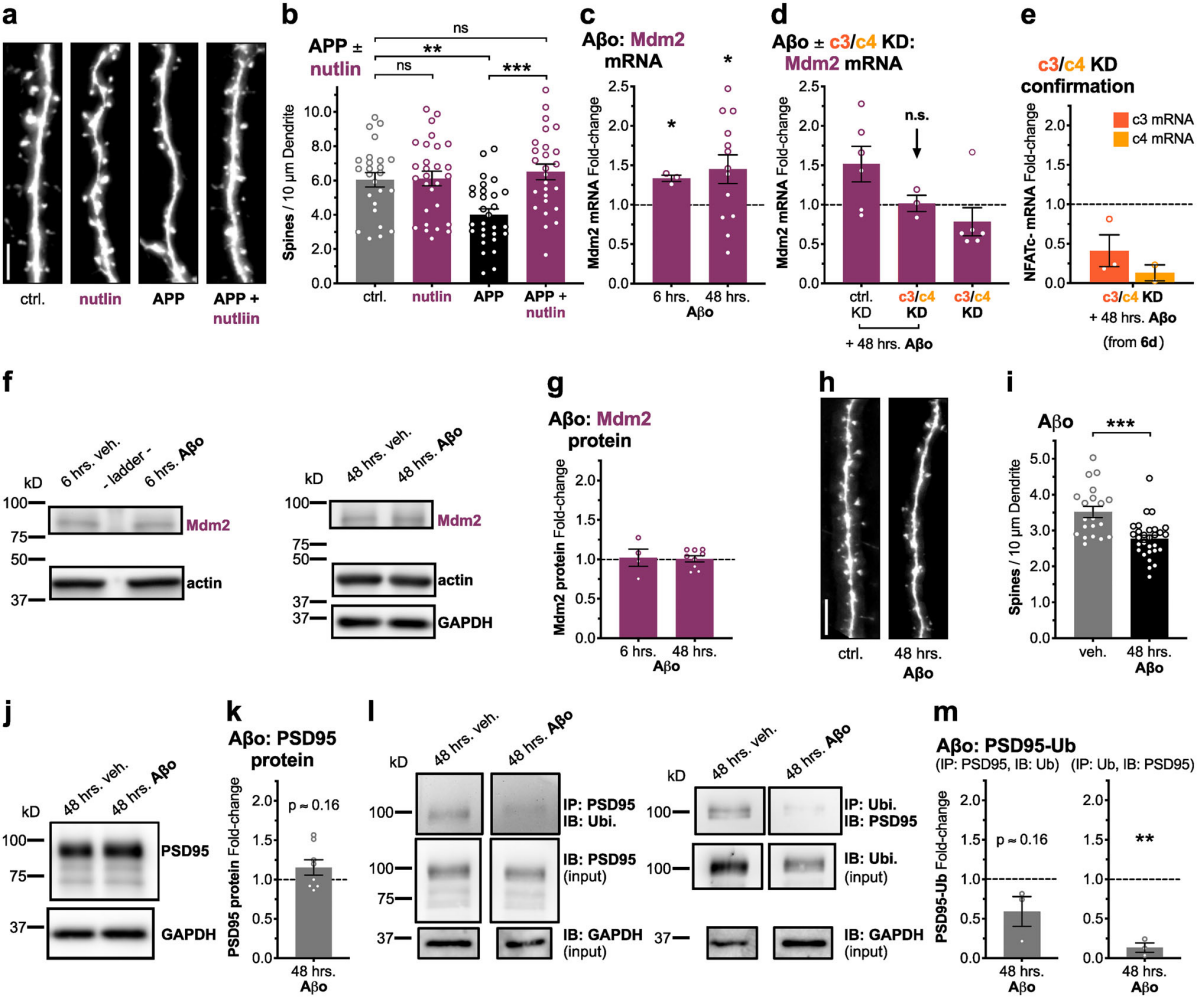
APP overexpression-induced spine loss requires Mdm2 activity, and Aβ-induced Mdm2 transcriptional upregulation requires NFAT. ***a***, Representative dendrite images from dissociated rat hippocampal neurons expressing mCh (5 μm scale bar). Cells were co-transfected at DIV 12–14 with mCh and either control or APP plasmids, allowed to overexpress for 72 h with nutlin or vehicle (H_2_O) present, and fixed at DIV 15–17. ***b***, Mean dendritic spines/10 μm dendrite (±SEM) for ctrl and APP overexpression conditions, with nutlin or vehicle (H_2_O). 2 μM racemic nutlin-3, *n* = 25–29 cells per condition, *N* = 3 cultures. **p < 0.01, ***p < 0.001 by one-way ANOVA with Sidak's correction. ***c***, Mean fold-change (±SEM) in Mdm2 mRNA by RT-qPCR for rat hippocampal cultures treated with bath-applied 500 nM soluble Aβo, relative to exposure-time-matched vehicle-treated (PBS/DMSO) controls. Cultures were treated with Aβo or vehicle for either 6 or 48 h prior to RNA harvest at DIV 15–16. *n* = 3–13 samples per time point, *N* = 3–6 cultures. *p < 0.05 by one-sample *t* test with theoretical mean of 1.0. ***d***, Mean fold-change (±SEM) in Mdm2 mRNA for control- and knockdown-infected Aβo-treated rat hippocampal cultures, relative to control-infected vehicle-treated controls. Cultures were infected at DIV 10–11 and were treated with 500 nM Aβo or vehicle for 48 h prior to RNA harvest at DIV 15–16. *n* = 3–6 samples per condition, *N* = 3 cultures. To quantify Mdm2 mRNA fold-change in response to NFATc3/c4 double KD alone, double KD samples from Figure 3*d* were rerun for Mdm2 and quantified here. *n* = 6 samples, *N* = 3 cultures. ***e***, Mean fold-change (±SEM) in NFAT mRNA for knockdown-infected Aβo-treated cultures from panel ***d***, confirming successful knockdown. *n* = 3 samples, *N* = 3 cultures. *p < 0.01$\bar{6}$ by one-sample *t* test with theoretical mean of 1.0 and Bonferroni's correction (*p *< *α*/3, *α* = 0.05). ***f***, Representative Western blots for protein lysates of rat hippocampal neuron cultures treated in parallel to those used for RT-qPCR in panel ***c***. Cultures were treated with Aβo or vehicle for either 6 or 48 h prior to protein harvest at DIV 15–16. ***g***, Mean fold-change (±SEM) in Mdm2 total protein by Western blot for Aβo-treated cultures, relative to their exposure-time-matched vehicle-treated controls. *n* = 4–9 samples per time point, *N* = 3–4 cultures. ***h***, Representative dendrite images from dissociated rat hippocampal neurons expressing EGFP (5 μm scale bar). Cells were transfected at DIV 12–14 with EGFP and treated with 500 nM bath-applied Aβo or vehicle for 48 h prior to fixation. ***i***, Mean dendritic spines/10 μm dendrite (±SEM) for veh. and 48 h Aβo-treated conditions. *n* = 20–29 cells per condition, *N* = 3 cultures. ***p < 0.001 by two-tailed, unpaired *t* test. ***j***, Representative Western blots for protein lysates of rat hippocampal neuron cultures treated with Aβo or vehicle for 48 h prior to protein harvest at DIV 15–16. ***k***, Mean fold-change (±SEM) in PSD95 total protein by Western blot for Aβo-treated cultures, relative to their exposure-time-matched vehicle-treated controls. *n* = 8 samples from *N* = 7 total cultures. ***l***, Representative Western blots for protein lysates of rat hippocampal neuron cultures treated with Aβo or vehicle for 48 h prior to protein harvest at DIV 15–16 followed by immunoprecipitation and immunoblotting of PSD95 and ubiquitin. PSD95 was immunoprecipitated (BioLegend, 810401) and samples were subsequently immunoblotted for ubiquitin (Santa Cruz, sc-8017). In parallel, ubiquitin was immunoprecipitated and immunoblotted for PSD95. A portion of each sample (labeled input) was reserved prior to immunoprecipitation and runs alongside the immunoprecipitated portion during immunoblotting to aid in quantification of normalized PSD95 ubiquitination levels. Immunoblotting for GAPDH provided an additional input loading control. ***m***, Mean fold-change (±SEM) in PSD95 ubiquitination by Western blot for 48 h Aβo-treated samples, relative to their exposure-time-matched vehicle-treated control samples. *N* = 3 cultures. **p < 0.01 by one-sample *t* test with theoretical mean of 1.0.

Next, we sought to determine if the NFAT transcriptional target Mdm2 (Zhang et al., 2012) was involved in Aβ-induced dendritic spine elimination. Mdm2 is an E3-ubiquitin ligase, perhaps best known for regulating the activity of the p53 tumor suppressor family of transcription factors (Chene, 2003), but also regulates synaptic levels of PSD95 during LTD (Colledge et al., 2003) and was previously implicated in developmental synapse elimination (Tsai et al., 2017). First, we observed that application of the highly specific Mdm2 inhibitor nutlin-3 (Vassilev, 2004) (2 μM racemic, 1 μM effective) blocked 72 h APP overexpression-induced spine loss and preserved normal spine density (***p < 0.001 for APP + nutlin to APP, n.s. for APP + nutlin to ctrl., one-way ANOVA with Sidak's; Fig. 6*a,b*). In addition, we observed that Mdm2 mRNA was significantly elevated in rat hippocampal cultures following both 6 and 48 h exposures to 500 nM Aβo (*p < 0.05 for Mdm2 mRNA at 6 and 48 h, one-sample *t* test, theoretical mean 1.0; Fig. 6*c*). Critically, we then confirmed that this Aβ-induced upregulation of Mdm2 mRNA was indeed blocked by NFATc3/c4 double shRNA knockdown (*p < 0.01$\bar{6}$ for Mdm2 mRNA at 48 h with ctrl. KD, n.s. for Mdm2 mRNA at 48 h with c3/c4 KD, one-sample *t* test, theoretical mean 1.0, Bonferroni's *p *< *α*/3 and *α* = 0.05; Fig. 6*d,e*). Interestingly, although we observed Aβ-induced upregulation of Mdm2 mRNA, Mdm2 total cellular protein levels appeared unchanged following 6 and 48 h exposures to Aβo (n.s. for Mdm2 total protein at 6 and 48 h, one-sample *t* test, theoretical mean 1.0; Fig. 6*f,g*), perhaps indicating a role for NFAT-dependent mRNA upregulation in maintaining adequate levels of Mdm2 expression and/or a more local, compartmentalized requirement for Mdm2 in Aβ-induced spine loss that is obscured in whole-cell level measurements.

Next, in response to 48 h bath-applied Aβo we examined the following: (1) the degree of dendritic spine loss, (2) ubiquitination of PSD95, and (3) potential degradation of PSD95. Surprisingly, while 48 h Aβo exposure significantly reduced dendritic spine density in cultured rat hippocampal neurons (***p < 0.001 for Aβo to veh., unpaired two-tailed *t* test; Fig. 6*h,i*), PSD95 total protein did not decrease; rather it may even be increased, although this trend did not prove significant in our hands (n.s. *p* ≍ 0.16 for 48 h Aβo, one-sample *t* test, theoretical mean 1.0; Fig. 6*j,k*). In parallel, we performed immunoprecipitations of PSD95 and ubiquitin followed by immunoblotting for ubiquitin and PSD95, respectively (Fig. 6*l,m*). Not only did PSD95 ubiquitination not increase in response to 48 h Aβo exposure, but it instead was significantly reduced (**p < 0.01 for 48 h Aβo, one-sample *t* test, theoretical mean 1.0; Fig. 6*l,m*), at least when assessed by immunoprecipitating ubiquitin followed by immunoblotting for PSD95. Together, these data indicate that although Mdm2 activity is required for APP/Aβ-induced dendritic spine loss, targeting PSD95 for degradation is not likely the mechanism by which spine collapse and synapse loss are triggered.

## Discussion

**Figure 7.**
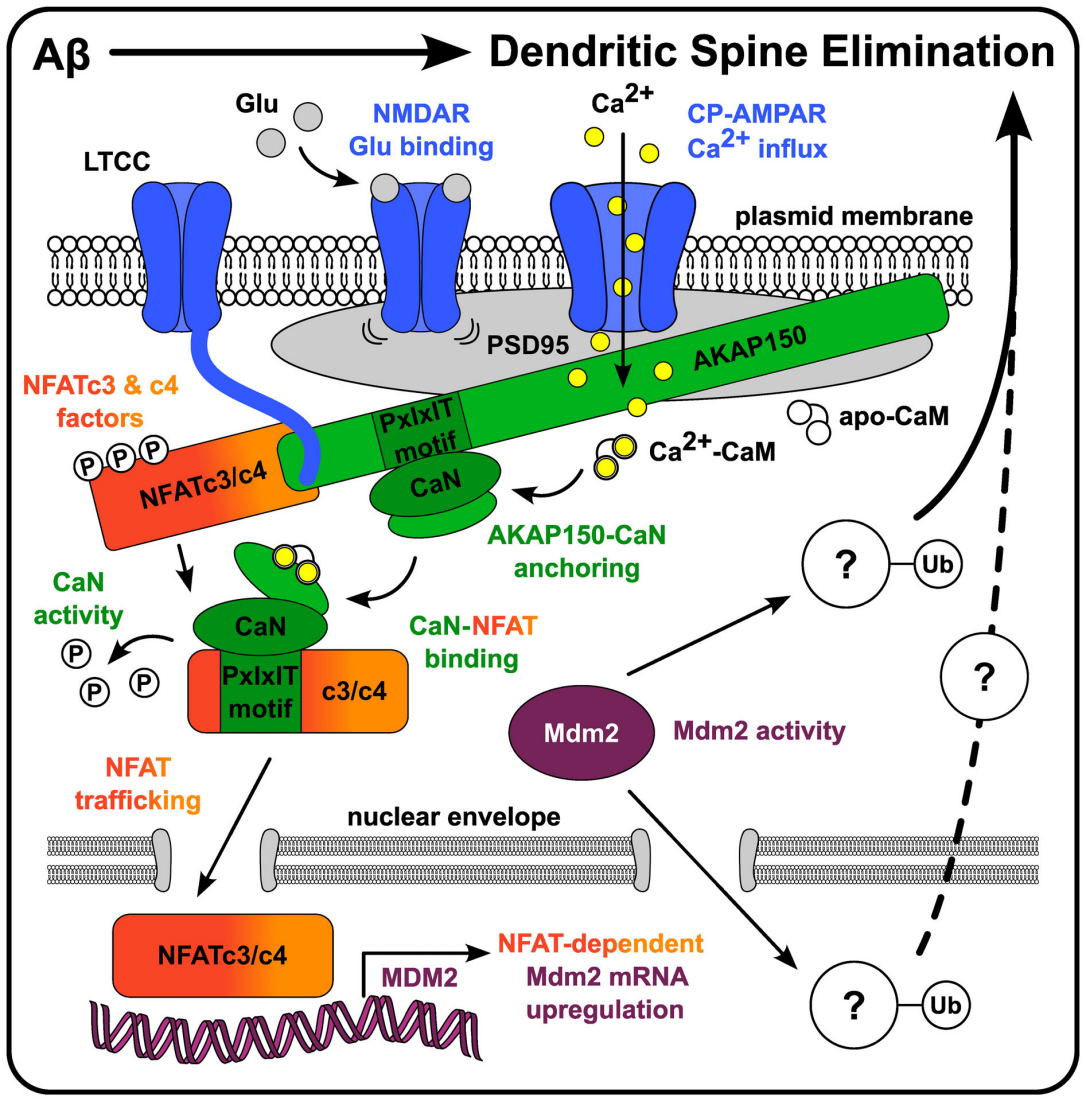
Diagram summarizing the CP-AMPAR-AKAP-CaN-NFAT-Mdm2 excitation-transcription coupling signaling pathway required for Aβ-mediated dendritic spine loss characterized in this study.

Here, we modeled APP proteolytic processing-mediated Aβ_42_ overproduction and subsequent Aβ-induced excitatory synapse dendritic spine loss in vitro by overexpressing an APP construct harboring multiple FAD mutations in dissociated rodent hippocampal neurons. In conjunction, we used a range of pharmacologic and genetic approaches to carefully interrogate the underlying postsynaptic signaling mechanisms required for the observed spine loss. Briefly, our results indicate that Aβ-induced dendritic spine loss requires the following: (1) nonionotropic NMDAR conformational signaling, (2) Ca^2+^ influx through CP-AMPARs, (3) AKAP150-CaN anchoring, (4) coupling to CaN-dependent NFATc3 and NFATc4 transcription factors, and (5) enzymatic activity of the downstream NFAT transcriptional target Mdm2. (See Figure 7 for a diagram summarizing these findings). We also overexpressed each of the two predominant hippocampal NFAT isoforms to reveal that NFATc3 overexpression and subsequent CaN-NFATc3 signaling are sufficient to recapitulate Aβ-induced spine loss, while overexpression of NFATc4 is not. Using soluble oligomers of synthetic Aβ_42_ peptide, we also reveal stark differences in Aβ-induced NFATc3 and NFATc4 nuclear enrichment, and importantly we uncover Aβ-induced, NFAT-dependent Mdm2 mRNA upregulation.

Our first observation that the NMDAR antagonist AP5 blocked APP overexpression-induced dendritic spine loss is unsurprising and consistent with prior studies where AP5 or similar antagonists that compete with glutamate binding to GluN2 subunits (e.g., CPP) demonstrated a requirement for NMDARs in Aβ-induced spine loss and inhibition of LTP (Shankar et al., 2007; Wei et al., 2010). Yet, the exact role of NMDARs in Aβ-induced synaptic dysfunction and spine loss has remained an open question. NMDAR Ca^2+^ influx is typically required for both LTP and LTD, and the magnitude and duration of the resulting rise in intracellular [Ca^2+^] is often a determinant of synaptic strengthening or weakening (Luscher and Malenka, 2012). In this regard, Aβo potently attenuates NMDAR Ca^2+^ influx (Sinnen et al., 2016), and low-level NMDAR-Ca^2+^ signaling is sufficient for LTD induction and to promote spine loss (Mulkey and Malenka, 1992; Shankar et al., 2007), hinting at least one potential role for regulation of NMDARs in Aβ-induced, LTD-like synaptic depression and spine loss. However, considerable evidence also indicates that glutamate-dependent, nonionotropic NMDAR conformational signaling, which is blocked by drugs like AP5 and CPP but preserved in the presence of pore blockers (e.g., MK801) and GluN1 glycine-site competitive antagonists (e.g., 7CK), is sufficient for functional and structural LTD (Nabavi et al., 2013; Stein et al., 2015) and also for Aβ-induced synaptic depression and spine loss (Kessels et al., 2013; Birnbaum et al., 2015). Furthermore, NMDAR conformational signaling, when isolated by MK801 or 7CK, appears sufficient to elicit functional and structural LTD even in response to LTP stimuli (Nabavi et al., 2013; Stein et al., 2015). Here, our findings that AP5 blocked APP overexpression-induced spine loss, while MK801 failed to do so, are consistent with this evolving view of NMDARs and indicate that glutamate-dependent NMDAR conformational signaling is indeed required for Aβ-induced dendritic spine loss. Moreover, our observation that MK801 independently drove spine loss indicates that NMDAR conformational signaling, isolated here by ionotropic blockade, is likely sufficient to drive spine loss in the presence of spontaneous activity alone. Altogether our findings bolster growing evidence that implicates NMDAR conformational signaling in both Aβ-induced spine loss and LTD-associated dendritic spine structural plasticity more generally. It should also be noted that although NMDAR-Ca^2+^ may not be strictly required for Aβ-induced synaptic depression or spine loss, it still stands to reason that, by preventing higher levels of postsynaptic Ca^2+^ signaling required for LTP, Aβ-induced attenuation of NMDAR-Ca^2+^ influx (Sinnen et al., 2016) may support LTD-like synaptic depression/spine shrinkage to promote Aβ-induced spine loss that is concomitantly driven by nonionotropic conformational signaling. Of note, while the MK801-driven spine loss we observed here was not significantly different from APP overexpression-induced spine loss in the presence of MK801, there is a trending difference in their means that barely failed significance testing (*p *≍ 0.06; Fig. 1*c,d*), suggesting that perhaps cooperative mechanisms are at work.

Additionally, it is well established that many posttranslational modifications required for Aβ-induced synaptic dysfunction and spine loss depend on Ca^2+^-CaM-dependent enzymes that are typically associated with synaptic plasticity (e.g., CaMKII, CaN) (Chen et al., 2002; Hsieh et al., 2006; Shankar et al., 2007; Opazo et al., 2018; Cook et al., 2019). This raises an important question about the potential source(s) of this Ca^2+^, since as investigated above NMDAR Ca^2+^ influx may not be strictly required. Our finding that NASPM, a CP-AMPAR pore blocker, prevented APP overexpression-induced spine loss partly addresses this question and comports well with existing evidence that implicates CaN regulation of CP-AMPARs in both normal synaptic plasticity (e.g., LTP, LTD, homeostatic plasticity) (Sanderson et al., 2012, 2016, 2018; Kim and Ziff, 2014) and Aβ-induced inhibition of LTP (Sanderson et al., 2021). Here, our data show that CP-AMPARs are further required for Aβ-induced dendritic spine loss. While this does shed light on certain aspects of Aβ-dependent signal transduction, it is unlikely that CP-AMPARs alone provide sustained Ca^2+^ signals over the duration of time required for spine loss to manifest and during which the activity of Ca^2+^-CaM-dependent enzymes such as CaN is required. Accordingly, we also tested for the potential involvement of LTCCs and observed that the dihydropyridine antagonist nimodipine failed to protect against APP overexpression-induced spine loss. However, this result aligns with previous reports that nifedipine, a similar dihydropyridine, failed to block NMDAR ion-flux-independent LTD isolated by the presence of 7CK (Nabavi et al., 2013). Although not explored here, other potential Ca^2+^ sources that could be engaged by Aβ to aberrantly drive CaN signaling include TRPM2 channels (Ostapchenko et al., 2015) and ryanodine receptor-mediated release from internal stores (Chakroborty et al., 2012; Zhang et al., 2023). Nevertheless, taken together, our results indicate that, in addition to nonionotropic conformational NMDAR signaling, Aβ-induced dendritic spine loss also requires ionotropic CP-AMPAR signaling.

As alluded to, CaN is required for NMDAR-dependent functional and structural LTD (Mulkey et al., 1994; Zeng et al., 2001; Zhou et al., 2004), as well as for Aβ-induced synaptic dysfunction and subsequent spine and synapse loss (Chen et al., 2002; Snyder et al., 2005; Hsieh et al., 2006; Shankar et al., 2007; Reese et al., 2008; Wu et al., 2010, 2012; Zhao et al., 2010). Specifically, CaN regulates endocytosis of both CI-AMPARs and CP-AMPARs at least in part by direct dephosphorylation of GluA1 subunits (Beattie et al., 2000; Lee et al., 2000; Man et al., 2007; Sanderson et al., 2012), and CaN promotes F-actin depolymerization by activation of slingshot–cofilin signaling (Zhou et al., 2004; Wang et al., 2005). Thus, our finding that CsA and FK506, which target CaN through similar yet distinct mechanisms, each block APP overexpression-induced spine loss is in line with these prior studies. However, a wealth of evidence has also shown that CaN-dependent endocytosis of CI-AMPARs and CP-AMPARs not only requires CaN activity but also requires synaptic targeting and localization conferred by anchoring to the postsynaptic signaling scaffold AKAP150 (Beattie et al., 2000; Bhattacharyya et al., 2009; Jurado et al., 2010; Sanderson et al., 2012, 2016). Importantly, AKAP150-CaN anchoring also regulates the synaptic incorporation and removal of CP-AMPARs that controls both LTD (Sanderson et al., 2012, 2016) and Aβ-induced inhibition of LTP (Sanderson et al., 2021). As AMPAR endocytosis precedes and is required for Aβ-induced spine loss (Hsieh et al., 2006; Zhao et al., 2010), we were thus not surprised that genetic deletion of the AKAP150-CaN anchoring PxIxIT motif blocked APP overexpression-induced dendritic spine loss.

However, NFAT transcriptional signaling is an additional pathway downstream of AKAP-anchored CaN that has also previously been implicated in Aβ-mediated spine loss and AD (Abdul et al., 2009, 2010; Wu et al., 2010; Hudry et al., 2012). Accordingly, disruption of this key postsynaptic excitation-transcriptional signaling pathway could also explain the protection against APP/Aβ-driven spine loss observed for AKAP150ΔPIX neurons. Importantly, either acute genetic disruption of AKAP-CaN anchoring using an RNAi knockdown-replacement approach in rat neurons or chronic genetic disruption AKAP-CaN anchoring in neurons from AKAP150ΔPIX mice impairs both NFATc3 and NFATc4 activation in response to a variety of neuronal stimuli that engage NMDARs, AMPARs, and LTCCs (Oliveria et al., 2007; Li et al., 2012; Murphy et al., 2014; Wild et al., 2019). As mentioned above, previous studies implicating CaN-NFAT signaling in Aβ-mediated spine loss (Hudry et al., 2012) relied on overexpression of protein fragments and peptides, such as the PVIVIT peptide, that contain the CaNA subunit consensus docking motif PxIxIT, which is also found in many other CaN scaffolding proteins (e.g., AKAP150), inhibitors (e.g., RCAN1), and substrates in addition to NFAT, including the LTD-required, CaN-regulated nuclear transcription factor CRTC1 (Li et al., 2011; Pan et al., 2021). Thus, it is possible that the ability of PxIxIT-site competitor overexpression to prevent Aβ-mediated spine loss in previous work, even when targeted to the nucleus, could have been due to disruption of CaN regulation of other targets in addition to or instead of NFAT (Hudry et al., 2012). Previous studies also only examined how Aβ might alter the nuclear localization of one of the two major neuronal NFAT isoforms, NFATc4 (Wu et al., 2010; Hudry et al., 2012). Accordingly, here we used RNAi as well as overexpression approaches to examine possible roles more directly and specifically for both NFATc3 and NFATc4 in Aβ-mediated spine loss.

While our results using RNAi knockdown indicate potentially overlapping/redundant roles of NFATc3 and NFATc4 in Aβ-mediated spine loss, a limitation of these experiments is that the level of mRNA knockdown we achieved was at best 60–70% for each NFAT isoform and with single isoform knockdown we typically also observed mRNA upregulation for the other NFAT isoform that was not targeted (T.P.M and M.L.D. unpublished observations). In contrast, our results demonstrating that NFATc3, but not NFATc4, overexpression is able to drive spine loss that mimics and occludes spine loss produced by APP is consistent with a more prominent, and even potentially dominant, role for NFATc3 in response to Aβ than previously thought. While a previous study found that overexpression of a truncated form of NFATc4 that is constitutively localized to the nucleus, due to removal of all the docking and phosphorylation sites required for CaN regulation, was also able to drive dendritic spine loss, due to nature of this truncation there is no way to link the endpoint of spine loss specifically to activity-dependent CaN regulation of NFATc4 (Hudry et al., 2012). In contrast, by introducing just two Ile-Ala mutations into the PxIxIT motif in NFATc3, to reduce CaN binding affinity and impair nuclear translocation, we were able prevent the spine loss observed with WT NFATc3 expression, thus linking this phenotypic convergence with APP specifically to activity-dependent CaN regulation of NFATc3. Likewise, our analysis of the nuclear/cytoplasmic occupancy ratio for NFATc3 and NFATc4 in the presence of spontaneous activity with Aβo present compared to when Aβo is not present and when neuronal activity is silenced with TTX also indicates a greater role for NFATc3 in mediating signaling to the nucleus in hippocampal neurons in response to Aβ. Importantly, we also identified a role for CP-AMPARs in driving this observed Aβ-specific increase in NFATc3 nuclear occupancy.

Finally, using a candidate approach we were able to identify an NFAT target gene, the E3-ubiquitin ligase Mdm2, that was transcriptionally upregulated following Aβ exposure and whose enzymatic activity was required for APP/Aβ-driven spine loss. While Mdm2 was previously identified as an NFAT-regulated gene in cancer cells, where it plays a prominent role in regulating degradation of the pro-apoptotic, tumor suppressor transcription factor p53 (Chene, 2003; Zhang et al., 2012), our results here indicate that it is also NFAT-regulated in neurons. We were initially attracted to Mdm2 as a potential neuronal NFAT-regulated gene that could be involved in Aβ-mediated spine loss because of its previous links to LTD (through mediating PSD95 ubiquitination, removal from synapses, and degradation (Colledge et al., 2003)), and also to activity-dependent developmental synapse elimination mediated by MEF2 (which is another CaN-activated transcription factor (Tsai et al., 2017)). Accordingly, we found Aβo application increased Mdm2 mRNA expression and that NFATc3/c4 double knockdown prevented this increase. Furthermore, using the specific Mdm2 inhibitor nutlin-3 we found that the E3-ubiquitin ligase activity of Mdm2 was required for APP/Aβ-driven dendritic spine loss. However, we found that Aβo exposure did not lead to PSD95 degradation and instead resulted in decreased PSD95 ubiquitination. Thus, surprisingly, Mdm2-Ubq-mediated degradation of PSD95 is not likely involved in Aβ-mediated spine loss.

Interestingly, we did not detect any increase in the level of Mdm2 total protein expression by immunoblotting neuronal whole-cell extracts following Aβo treatment despite mRNA upregulation. This result suggests a few possibilities. While some Mdm2 is present in dendrites, consistent with its known synaptic regulatory roles, the majority of Mdm2 is found in the nucleus in the cell body, consistent with its prominent transcriptional regulatory roles (Colledge et al., 2003; Tsai et al., 2017). Thus, if the Aβ-mediated increase in Mdm2 mRNA expression is primarily required to increase dendritic expression levels of Mdm2 (perhaps through local mRNA translation?), any such local increase in Mdm2 protein in dendrites may be obscured in immunoblotting experiments by the much greater signal contributed by Mdm2 in the neuronal nucleus or even other in other cell types present in the culture, such as astrocytes. Although not mutually exclusive with the above possibility, an equally plausible, alternate explanation is that the upregulation of Mdm2 mRNA expression is required to just maintain some crucial level of total Mdm2 protein expression and catalytic activity that is required for Aβ-mediated synapse elimination, either through its activity in dendrites and/or in the nucleus through transcriptional regulation of p53 family transcription factors. Regardless, our finding that the Mdm2 inhibitor nutlin-3 completely prevents APP/Aβ-driven spine loss is consistent with a central role for Mdm3 E3 ligase activity in this process. However, we of course cannot rule out that one or more other NFAT-regulated gene products, in addition to Mdm2, may also be required for Aβ-mediated synapse elimination.

Overall, our findings presented here elucidate a novel synapse-to-nucleus signaling pathway engaged by Aβ to promote dendritic spine loss. More specifically, this pathway is activated by Ca^2+^ influx through CP-AMPARs and transduced to the nucleus by CaN-NFAT signaling that is coordinated by the postsynaptic scaffold protein AKAP150 to transcriptionally upregulate the E3-ubiquitin ligase, Mdm2, whose catalytic activity is in turn required for spine loss. In the future, it will be important to identify additional transcriptional targets of NFAT regulated by Aβ that may also be required for spine loss, how Mdm2 protein levels are regulated by Aβ, and what Mdm2 substrates are required for Aβ-mediated spine loss, including potentially other synaptic proteins and/or nuclear proteins.

## Synthesis

Reviewing Editor: Tod E Kippin, University of California Berkeley Decisions are customarily a result of the Reviewing Editor and the peer reviewers coming together and discussing their recommendations until a consensus is reached. When revisions are invited, a fact-based synthesis statement explaining their decision and outlining what is needed to prepare a revision will be listed below. The following reviewer(s) agreed to reveal their identity: NONE.

Synthesis

Both reviewers found merit in your work as you can appreciate in their comments below. However, they suggest the need for additional experiments to validate your results, including the use of a different model such as WT-APP over expression or ideally oligomeric A-beta.

Reviewer's comments

Reviewer#1

This work seeks to delve deeper into the mechanisms that drive dendritic spine loss in response to elevation of oligomeric Abeta, which has been established to reduce dendritic spine densities in hippocampal neurons. It is likely that this type of mechanism is responsible for reduced excitatory synapse number in Alzheimer's disease individuals. The authors dominantly use overexpression and some genetic models to elucidate mechanisms of spine loss that include CP-AMPARs, the phosphatase CaN, AKAP150, NFAT and Mdm2. Understanding these pathways could be relevant for the treatment of AD. While the manuscript does a fine job of elucidating these factors individually, the authors fail to determine if the signaling pathways are functioning in a linear pathway and if they are in fact due to increases in oligomeric Abeta and not related to Abeta42-independent effects of overexpressing APP.

Experiment requests:

The use of APP overexpression to drive the production of oligomeric Abeta works well, however as stated above, it is possible that APP overexpression drives aspects of dendritic spine loss that are Abeta independent, which is an issue that must be resolved in this paper. Therefore, it will be critical to perform experiments with pharmacological agents and genetic manipulations using oligomeric Abeta. This is especially important to link changes in Mdm2 transcript that may be occurring upon APP overexpression but were never tested.

-It is clear that blocking Mdm2 prevents the dendritic spine loss, and this is most likely due to posttranslational regulation of Mdm2 substrates as opposed to changes in Mdm2 mRNA. The authors should determine if PSD-95 levels are stabilized upon treatment with Nutlin-3 as this would be the most likely target that would reduce dendritic spine density upon overexpression of oligomericAbeta.

-All of the pharmacological agents used in this paper are meant to block synapse to nucleus transmission. Therefore, it is expected that these agents may also block the movement of NFATc3 into the nucleus to drive gene expression changes that would lead to dendritic spine loss. This should be tested.

-It will be important to determine if APP overexpression alters NFAT phosphorylation to link CaN signaling with its movement to the nucleus.

Technical and qualitative issues with the data presented:

-Expression of APP is not validated in neurons co-transfected with APP and mCherry. This is a critical control for all experiments.

-Figure 3: knockdown of c3 and c4 shows a reduction in mRNA but one must also show a loss of protein for these studies.

-The nuclear/cytoplasmic changes of NFAT in Figure 5a are unconvincing. Moreover, the DAPI nuclei marker should be shown in all figures. All of the NFAT localization studies rely on overexpression. It will be essential to show movement of endogenous NFAT in response to activity, Abeta oligomerization, and APP overexpression.

-Are oligomeric Abeta changes in Mdm2 mRNA NFAT-dependent?

Comments related to experimental details:

-Concentrations for pharmacological agents are reported but the duration of treatment for pharmacological agents also needs to be reported. Vehicle control conditions for each agent must also be defined (DMSO, water, ethanol, etc.)

-The authors should report in the methods if apical or basal 2o and 3o dendrites were targeted.

Minor comments:

Line 114: "E3-ubiquirin" should be "E3-ubiquitin"

Reviewer#2

In this manuscript, authors delve into the molecular grounds of Aβ synaptotoxic effects using in vitro pharmacologic and genetic tools. They show that APPswe-ind overexpression, which increases Aβ production, reduces spine density in hippocampal primary cultures of rats. This spine loss is blocked by AP5 (NMDA competitive antagonist), NASPM (Calcium-permeable AMPAR pore blocker) and MK801 (NMDA calcium channel blocker), but not nimodipine (L-type voltage-gated calcium channel blocker). It is also block by the inhibition of calcineurin and/or calcineurin-AKAP150 interaction (using CsA and FK506 inhibitors and genetic tools, respectively), and the combined knock-down of NFATc3 and NFATc4 (two transcription factors downstream of Calcineurin signaling).

Authors also show that NFATc3 overexpression and recruitment to the nucleus induces a significant loss of spines, and that the inhibition of the NFAT-transcriptional target Mdm2 (using nutlin3) is sufficient to block the spine loss induced by APPswe-ind overexpression.

The article is well written and pleasant to read. As the authors properly acknowledge, Aβ effects on NMDA and AMPA receptors and calcium-calcineurin-AKAP150 signaling were previously described. It was also known the activation of NFAT and the downstream Mdm2. However, despite its limited novelty, the manuscript still provides new and valuable data of potential interest to experts in the field.

The manuscript would benefit from additional experimental evidence:

It would be desirable to test whether wild-type APP overexpression induces similar effects than APPswe-ind. It will allow to disentangle APP overexpression from Aβ overproduction effects (Figures 1-6, for spine density and pathways). Aβ-mediated additional evidence could also be obtained by assaying Aβ oligomer effects.

Evidence for Mdm2-dependent APPswe-ind overexpression-induced spine loss is limited to nutlin3 treatment. It would be desirable to complement these experiments with genetic manipulations (e.g., shRNA) and/or alternative approaches.

A deeper analysis on Mdm2 protein levels using synaptic fractions or alternative methods would also increase the biological significance of the results.

## Author Response

Point-by-point response to previous review of eN-NWR-0175-23R1 "Amyloid-β-induced dendritic spine elimination requires Ca2+-permeable AMPA receptors, AKAP-Calcineurin-NFAT signaling, and the NFAT target gene Mdm2" We thank the reviewers for their constructive feedback and overall positive comments on our revised manuscript and the consensus that this study would be acceptable for publication in eNeuro with minor revisions. We feel that the revisions we have made should adequately address the most recent comments and make the manuscript now acceptable for publication in eNeuro. Revisions to the manuscript text from R1 are indicated in blue and revisions for the current R2 submission are indicated in red.

Please note that we deliberately include in some cases one and two condition bar graphs where the individual data points are overlayed as a scatter blot on top of the mean {plus minus} SEM bar graph. We feel that this way of representing of the actual distribution of the data (even for single condition) is far more rigorous than just mentioning mean values {plus minus} SEM or SD in the text. Thus, we strongly feel that our figures should be presented with these one and two condition bar graphs included, despite seeming to conflict with the eNeuro recommended figure formatting guidelines.

Reviewer # 1 Overall, the authors have addressed revisions to the best of their ability, providing convincing mechanistic evidence of a synaptic to nuclear pathway that drives dendritic spine loss following the production of Abeta oligomers.

Minor edits: "This work would benefit by providing a schematic of the mechanisms outlined in the paper to simplify for the broad readership." We now provide a visual abstract highlighting our major findings that includesa schematic of the signaling mechanisms reported in the study. "Figure 6j could benefit by providing a loading control (e.g. GAPDH) for the input samples to ensure the same amount of material was inputted for each condition.... Recommend switching figures 6l and 6m with 6j and 6k." The order of Figure panels 6j,k, and l,m has been switched as requested. In addition, we stripped and re-probed the blots with anti-GAPDH as requested and now provide these input loading controls in new Fig. 6l. "Check grammar on line 456." We reworded this section to be more clear and fix grammatical errors "Provide appropriate references for line 622" Hudry et al. 2012 was added here and in another part of this section (along with Wu et al., 2010) to ensure appropriate referencing of the prior literature.

Reviewer #2 "The revised version of the manuscript resolves the concerns raised in the previous version." No response needed.
